# Comparative study of antibiotic resistance and virulence profiles of *exoU* and *exoS Pseudomonas aeruginosa* isolates from urinary tract infections and microbial keratitis in Bangladesh and India

**DOI:** 10.1007/s11274-026-05320-2

**Published:** 2026-10-12

**Authors:** Tanzina Akter, Shiful Islam, Abrar Maswood Haider, Rifaquat Ahmed, Kaniz Fatema, Md. Mozammel Hoque, Fiona Stapleton, Mark Willcox

**Affiliations:** 1 School of Optometry and Vision Science, Faculty of Medicine and Health, University of New South Wales (UNSW), Sydney, NSW 2052 Australia; 2 Microbial Biotechnology Division, National Institute of Biotechnology (NIB), Dhaka, 1349 Bangladesh; 3 Department of Biotechnology, Faculty of Natural Science, Norwegian University of Science and Technology, Trondheim, 7034 Norway; 4 Department of Microbiology, Primeasia University, Banani, Dhaka, 1213 Bangladesh; 5 Department of Clinical and Molecular Medicine, Faculty of Medicine and Health Sciences, Norwegian University of Science and Technology, Trondheim, 7034 Norway

**Keywords:** *Pseudomonas aeruginosa*, *exoU*, *exoS*, Urinary tract infection, Microbial keratitis, Antibiotic resistance, Virulence factors

## Abstract

*Pseudomonas aeruginosa* is an important opportunistic pathogen in urinary tract infections (UTIs) and microbial keratitis (MK), but differences in resistance and virulence between *exoU* and *exoS P. aeruginosa* isolates across regions and infection sites remain poorly understood. This study compared antibiotic resistance and virulence profiles of *exoU* and *exoS P. aeruginosa* isolates from Bangladeshi and Indian UTIs, and from Indian UTIs and MK collections. A total of 23 Bangladeshi UTI isolates were analyzed by broth microdilution for susceptibility to ciprofloxacin, levofloxacin, gentamicin, and tobramycin, and by whole-genome sequencing (WGS) to identify resistance-associated mutations, acquired resistance genes, and virulence factors. These WGS data were compared with 15 publicly available Indian UTI genomes, while the Indian UTI genomes were compared with 19 publicly available Indian MK genomes. In the Bangladeshi UTI cohort, *exoU* isolates showed greater resistance to fluoroquinolones and aminoglycosides than *exoS* isolates. Comparative analysis of Bangladeshi versus Indian UTI isolates and Indian UTI versus MK isolates showed that *exoU* isolates carried a burden of resistance mutations, including enrichment of GyrA Thr83Ile, ParE Asp533Glu, MexR Val126Glu, MexB Ser1041Glu, and MexC Ala262Glu. Aminoglycoside-associated substitutions FusA2 Gln161Leu and ParR Leu153Arg and Ser170Asn were more common in *exoU* UTI isolates. Acquired resistance genes showed *aac(6′)-II* and *aac(3)-Id* were linked to Bangladeshi UTI, *rmtF* associated with Indian UTI, and *crpP* confined to MK *exoU* isolates. Virulence profiling showed *exoU* enrichment in *pldA*, *pvdY*, and *csrA*, whereas *exoS* was associated with *pilC*, *pilV*, *pilW*, *pilY2*, and *pilA*. Collectively, these results indicate that *exoU* and *exoS* isolates differ in both their resistance and virulence profiles, while also showing evidence of geographic and infection-site-specific adaptation.

**Supplementary Information:**

The online version contains supplementary material available at https://doi.org/10.1007/s11274-026-05320-2.

## Introduction

*Pseudomonas aeruginosa* is an opportunistic bacterium of clinical significance, responsible for a wide range of infections in different parts of the body including pneumonia, bloodstream infection, urinary tract infections (UTIs), and corneal infections (de Bentzmann and Plesiat 2011; Qin et al. 2022). The role of the bacteria in nosocomial infection is particularly important because these infections can result in further complications in immunocompromised and hospitalized patients (Obritsch et al. 2005). Globally, *P. aeruginosa* is responsible for approximately 7–10% of hospital-acquired UTIs, especially among patients with prolonged catheterization or underlying comorbidities (Lamas Ferreiro et al. 2017). In Bangladesh, hospital-based studies suggest an increasing isolation rate of multidrug-resistant *P. aeruginosa* from UTIs ranging from 4% to 6% (Sanjee et al. 2017; Sharmin et al. 2021). Though there are no data on national level surveillance, comparable large-scale studies from the neighboring country India indicates that the burden of *P. aeruginosa* in UTIs is about 6–7% (Kalal and Nagaraj 2016; Bhargava et al. 2022). Conversely, *P. aeruginosa* remains as one of the most significant causative microbes in microbial keratitis (MK), a severe corneal infection, especially among contact lens users and patients with corneal trauma (Badger-Emeka et al. 2024). Studies suggest that *P. aeruginosa* accounts for 17% of the isolates causing MK globally (Singh et al. 2026) and responsible for more than two-thirds of contact lens-related keratitis cases (Pinna et al. 2008; Stapleton 2023).

*P. aeruginosa* relies on a range of virulence factors which include adhesins, toxins, motility structures, enzymes, quorum-sensing, biofilm-formation capacity, bacterial secretion systems (type 1 secretion system to - type 6 secretion system) among others to cause severe infection inside the host cell (Veetilvalappil et al. 2022). Among these, the type 3 secretion system (T3SS) plays a central role functioning as a molecular syringe, which directly injects effector proteins inside the host cells disrupting host cellular functions through cytotoxicity, immune invasion and modulation of host signaling pathways (Gellatly and Hancock 2013; Horna and Ruiz 2021; Akter et al. 2026a). This T3SS has four effector proteins (ExoS, ExoT, ExoU and ExoY) which have different enzymatic activities and specific roles in pathogenesis (Horna and Ruiz 2021; Akter et al. 2026a). The bacterial strains positive for *exoU* are highly cytotoxic as they possess potent phospholipase A_2_ activity causing host cell lysis and extensive tissue damage (Phillips et al. 2003). The expression of this effector protein has been linked to fulminant disease and high mortality in animal models and clinical studies, showing the severity of this strain (Finck-Barbancon et al. 1997; Allewelt et al. 2000; Sawa et al. 2014; Pena et al., 2015). The ExoS effector, in contrast, disrupts the host cell cytoskeletal dynamics for intracellular invasion promoting survival, dissemination, immune evasion and modulation of cellular function (Rao et al. 2021; Wood et al. 2023). These functions are mediated by the ADP-ribosyltransferase and GTPase that facilitate such mechanisms (Wood et al. 2023). *exoU* and *exoS* genes are often reported to show a largely mutually exclusive distribution among clinical isolates (Shaver and Hauser 2004); however, co-detection of the two genes has been reported in some clinical isolate collections (Idris et al. 2012; Horna et al. 2019). The prevalence of *exoS* and *exoU* genes in *P. aeruginosa* varies by infection site and clinical setting. UTI isolates show greater heterogeneity, with *exoU* and *exoS* detected in 26.9% and 16.4% of 134 isolates in one study (Firouzi-Dalvand et al. 2016). In pediatric UTI isolates, *exoU* was reported in 19.2% and *exoS* in 92.3%, indicating that these categories were not mutually exclusive in that study (Pobiega et al. 2016) while carbapenem-resistant urinary isolates were predominantly *exoU* positive strain (94.8%) (Park and Koo 2022). In contrast, in MK, *exoS* is more common overall, being detected in 71% of isolates, whereas *exoU* was found in 29% (Akter et al. 2025). However, contact lens-related MK is more strongly associated with *exoU*-positive strains (Choy et al. 2008). While the ExoU and ExoS effectors of *P. aeruginosa* are linked to distinct pathogenic strategies and are known to influence the severity and clinical outcomes of MK (Sato et al. 2003), their contribution to UTI pathogenesis remains poorly understood. An in vitro bladder epithelial cell model showed that *exoU* and *exoS P. aeruginosa* strains did not differ significantly in intracellular persistence, although *exoU*-producing strains elicited a stronger early IL-1β response, indicating potentially greater cytotoxic potential rather than a clearly more severe UTI phenotype (Newman et al. 2022). Despite this observation, the mechanistic roles of *exoU* and *exoS* in UTI pathogenesis have not been directly established, even though both genes have been identified disproportionately in *P. aeruginosa* isolates from UTIs cases (Pobiega et al. 2016; Park and Koo 2022).

Timely intervention is crucial to stopping the rapid progression of diseases. Treatment of *P. aeruginosa* infections requires targeted antimicrobial therapy guided by culture and susceptibility results due to the organism’s intrinsic resistance and frequent multidrug resistance. In Bangladesh, treatment options for UTIs include fluoroquinolones (ciprofloxacin and levofloxacin), aminoglycosides, nitrofurantoin, and carbapenems such as meropenem, depending on the type of UTI (DGHS 2021). Moreover, several studies conducted in Bangladesh have demonstrated that *P. aeruginosa* isolates from UTIs patients are susceptible to aminoglycosides, particularly gentamicin and tobramycin, and fluoroquinolones, including ciprofloxacin and levofloxacin, suggesting that these agents remain effective treatment options against *P. aeruginosa* (Akter et al. 2016; Sanjee et al. 2017; Abedin et al. 2022). In case of MK, topical antibiotic therapy is the mainstay, with broad-spectrum fluoroquinolone or fortified combinations of aminoglycosides such as gentamicin, tobramycin and cephalosporins such as ceftazidime are used as initial empiric therapy (Arun and Georgoudis 2024). However, *P. aeruginosa* isolated from MK in India is becoming increasingly common (Joseph et al. 2023).

In *P. aeruginosa*, fluoroquinolone resistance is primarily mediated by mutations in the quinolone resistance-determining regions (QRDRs) of GyrA, GyrB, ParC, and ParE, together with overexpression of major multidrug efflux pumps such as MexAB-OprM, MexCD-OprJ, MexEF-OprN, and MexXY-OprM, which is often associated with SNPs in regulatory genes including *mexR*,* nalC*,* nalD*,* nfxB*,* mexT*,* mexS*, and *mexZ* (Jalal et al. 2000; Chávez-Jacobo et al. 2018; López et al. 2022; Akter et al. 2026c). Acquired resistance determinants such as *crpP* and *qnrVC1* may further reduce fluoroquinolone susceptibility (Chávez-Jacobo et al. 2018; Akter et al. 2026c). Aminoglycoside resistance in *P. aeruginosa* is mainly associated with aminoglycoside-modifying enzymes, increased efflux via MexXY-OprM, and SNPs in genes involved in its regulation or target-associated pathways, including *mexZ*,* armZ*,* parRS*, and *fusA* (Poole 2005; Atassi et al. 2023; Akter et al. 2025, 2026c). Although *exoU* and *exoS* are primarily virulence-associated genes, isolates carrying these genes may also differ in their antimicrobial resistance profiles. Previous studies have reported a greater association of *exoU*-positive strains with fluoroquinolone and aminoglycosides resistance in MK isolates (Subedi et al. 2018a; Akter et al. 2025), suggesting that virulence genotype and resistance determinants may be linked within particular *P. aeruginosa* lineages.

UTIs and MK represent anatomically and immunologically distinct clinical niches, yet *P. aeruginosa* can colonize and cause severe disease in both. Previous studies have examined the resistance and virulence characteristics of *P. aeruginosa* in UTIs and MK separately (Obritsch et al. 2005; Mittal et al. 2009; Khan et al. 2020; Akter et al. 2025, 2026d). Moreover, *exoU*- versus *exoS*-associated differences in antibiotic resistance and virulence determinants have been investigated primarily among MK isolates (Akter et al. 2025, 2026b, d). However, no study to date has investigated these differences between *exoU*- and *exoS*-positive UTI isolates from Bangladesh and India. In addition, although *exoU/exoS* distributions have been described independently in UTI and keratitis isolates, direct comparisons of resistance and virulence profiles across these infection sites remain limited.

To address these gaps, the present study included 23 newly sequenced Bangladeshi UTI isolates and compared *exoU*- and *exoS*-associated antibiotic resistance and virulence profiles within UTI isolates. Geographic comparisons were performed between Bangladeshi and Indian UTI isolates, while infection-site comparisons were conducted between Indian UTI and MK isolates. Multilocus sequence typing (MLST) and core-genome phylogenetic analyses were also used to contextualize genotype-associated findings in relation to sequence type (ST) and clonal background. Thus, the principal novel contributions of this study are the genomic characterization of Bangladeshi UTI isolates, geographic comparison of UTI populations, direct UTI-MK comparison, and phylogenetic contextualization of *exoU/exoS*-associated findings. These analyses are expected to provide further insight into region-specific, infection-site-specific, and clonal differences in this increasingly drug-resistant pathogen.

## Methodology

### Sample collection

A total of 23 *P. aeruginosa* UTI isolates collected from Bangladesh in 2023 were used in this study (Supplementary Table 1). Each isolate was recovered from a different patient, and all samples were de-identified prior to use. No isolates were known to be associated with a common outbreak. The isolates were preserved at -80 °C in the microbial culture collection of the School of Optometry and Vision Science, UNSW Sydney, Australia, and were sub-cultured on nutrient agar (Oxoid Ltd., Basingstoke, Hampshire, UK) before analysis. Identification of the isolates was confirmed using the VITEK 2 automated system for Gram-negative bacteria (bioMérieux, Baulkham Hills, NSW, Australia) following the manufacturer’s instructions (Ling et al. 2001).

### Antibiotic susceptibility testing

Antimicrobial susceptibility testing was carried out as previously described in an earlier study (Akter et al. 2025). Briefly, ciprofloxacin, levofloxacin, gentamicin, and tobramycin were tested in sterile 96-well plates using two-fold serial dilutions in cation-adjusted Mueller-Hinton broth (MHB; Becton Dickinson and Company, Franklin Lakes, NJ, USA) over a concentration range of 0.25–5120 µg/mL. Overnight cultures were adjusted to approximately 1 × 10^5^ CFU/mL, combined with antibiotic dilutions in equal volumes, and incubated at 37 °C for 18–24 h. *P. aeruginosa* ATCC 27,853 was included as the quality-control strain for antimicrobial susceptibility testing. Minimum inhibitory concentration (MIC) values were defined as the lowest concentration preventing detectable growth at 660 nm (Wiegand et al. 2008; Akter et al. 2025). Susceptibility was interpreted according to CLSI M100, 28^th^ edition (2018) using the following breakpoints: ciprofloxacin ≤ 1, 2, and ≥ 4 µg/mL; levofloxacin ≤ 2, 4, and ≥ 8 µg/mL; gentamicin ≤ 4, 8, and ≥ 16 µg/mL; and tobramycin ≤ 4, 8, and ≥ 16 µg/mL for the susceptible, intermediate, and resistant categories, respectively. These criteria were retained to ensure consistency and direct comparability with the previously published study (Akter et al. 2025). Isolates classified as either intermediate or resistant were grouped as resistant for subsequent analyses.

### DNA extraction and genome sequencing and analysis of sequencing data

Genomic DNA was extracted from all 23 *P. aeruginosa* Bangladeshi UTI isolates using the DNeasy Blood and Tissue Kit (Qiagen, Hilden, Germany) according to the manufacturer’s instructions. Whole-genome sequencing was subsequently performed, and the resulting sequence data were deposited in the European Nucleotide Archive (ENA) under BioProject accession number PRJEB110861. Paired-end libraries were prepared using the QIAseq FX DNA Library Kit with the QIAseq FX DNA Library CDI Kit (Qiagen, Hilden, Germany), and sequencing was carried out on the Illumina NextSeq 1000 platform using 150 bp paired-end reads (PE150). Raw sequencing reads were quality-checked using FastQC v0.12.1 (Andrews 2010) and trimmed using Trimmomatic v0.39 (Bolger et al. 2014). De novo genome assembly was performed using Unicycler v0.4.8 (Wick et al. 2017). Assembly quality was assessed using QUAST v5.3.0 (Gurevich et al. 2013), based on genome size, GC content, number of contigs, and N50, while genome completeness was evaluated using BUSCO v6.0.0 (Tegenfeldt et al. 2025) in genome mode against the *pseudomonadales_odb12* lineage dataset. Only assemblies with ≥ 95% complete BUSCOs were retained for downstream analyses. Genome annotation was performed using Bakta v1.9.3 with default parameters (Schwengers et al. 2021). The presence of *exoU* and *exoS* was determined using BLASTn, with genes considered present at ≥ 95% sequence identity and ≥ 95% query coverage. Single-nucleotide polymorphisms (SNPs) were identified using Snippy v4.6.0 ((Seemann 2015); https://github.com/tseemann/snippy) with genotype-matched reference genomes: *exoU*-positive isolates were mapped against *P. aeruginosa* UCBPP-PA14 (RefSeq assembly GCF_000014625.1; chromosome NC_008463.1), whereas *exoS*-positive isolates were mapped against *P. aeruginosa* PAO1 (RefSeq assembly GCF_000006765.1; chromosome NC_002516.2). The predicted functional effects of amino acid substitutions were assessed using the SIFT (Sorting Intolerant From Tolerant) algorithm (Ng and Henikoff 2003), with scores ≤ 0.05 considered deleterious and scores > 0.05 considered tolerated. Acquired antimicrobial resistance genes were identified using ResFinder v4.7.2 (Bortolaia et al. 2020), applying thresholds of ≥ 90% nucleotide identity and ≥ 80% gene coverage. Virulence-associated genes were identified against the Virulence Factor Database (VFDB) (Zhou et al. 2025), using thresholds of ≥ 90% sequence identity and ≥ 80% coverage.

A total of 15 publicly available Indian *P. aeruginosa* genomes from UTIs were included in this study for comparative analysis (Supplementary Table 1). The Indian strains were obtained from the BioProjects PRJNA924087, PRJNA514245, PRJNA927155, PRJNA729627, PRJNA230869, PRJNA1074546, and PRJNA1102609. To enable comparison between UTI and MK isolates, 19 Indian MK-associated *P. aeruginosa* genomes from previously published datasets (NCBI BioProjects No: PRJNA590804 and PRJNA431326) were included in the present analysis (Subedi et al. 2018b; Khan et al. 2020) (Supplementary Table 1). These publicly available Indian genomes were downloaded as assembled genome sequences in FASTA format rather than as raw sequencing reads. Therefore, read trimming and de novo assembly were not performed for the Indian genomes. According to the associated NCBI metadata, these genomes were also generated using Illumina sequencing platforms, consistent with the sequencing technology used for the Bangladeshi UTI isolates. The quality and completeness of the downloaded assemblies were assessed using QUAST and BUSCO, respectively, followed by Bakta annotation and the same downstream analyses applied to the Bangladeshi isolates, including detection of *exoU*/*exoS*, identification of acquired antimicrobial resistance and virulence-associated genes, and SNP analysis using the same genotype-matched reference genomes.

### Multilocus sequence typing and phylogenetic analysis

STs were assigned with mlst v2.33.1 (Seemann: https://github.com/tseemann/mlst) using the PubMLST database (Jolley et al. 2018). For phylogenetic analysis, all 57 genomes, together with the *P. aeruginosa* PA14 reference genome (RefSeq assembly GCF_000014625.1; chromosome NC_008463.1), were mapped against the *P. aeruginosa* PAO1 reference (RefSeq assembly GCF_000006765.1; chromosome NC_002516.2) with Snippy v4.6.0 ((Seemann 2015); https://github.com/tseemann/snippy), and a core alignment was generated with snippy-core. Recombinant regions were removed with Gubbins v3.4.3 (Croucher et al. 2015), and variable sites were extracted with SNP-sites v2.5.1 (Page et al. 2016), resulting in a core-SNP alignment of 113,731 positions. A maximum-likelihood phylogeny was inferred with IQ-TREE v3.1.3 (Wong et al. 2026) under the GTR + F+ASC+R3 model and 1,000 ultrafast bootstrap replicates (Hoang et al. 2018). The tree was visualized and annotated with the Interactive Tree of Life (iTOL) (Letunic and Bork 2021), with tips annotated according to *exoU/exoS* genotype, ST, infection type, and country of origin.

### Statistical analysis

All statistical analyses were conducted using GraphPad Prism version 8.0.2 (263) (GraphPad Software, San Diego, CA, USA). Fisher’s exact test was used to assess differences in *exoU/exoS* distribution between cohorts and differences in SNP, acquired resistance gene, and virulence factor gene prevalence between *exoS*- and *exoU*-positive isolates. Spearman’s rank correlation was used to assess associations between MMR-gene SNP counts (*mutL*,* mutS*, and *uvrD*, individually and as the combined MMR SNP count) and the total number of AMR-associated SNPs per isolate. For associations between dichotomous variables, such as SNP presence/absence and antibiotic resistance status, the phi coefficient (φ) was used to quantify the strength of association, with statistical significance assessed using Fisher’s exact test. *P*-values were adjusted for multiple comparisons using the Benjamini–Hochberg (BH) procedure where applicable. An adjusted *p*-value of < 0.05 was considered statistically significant. Group-wise genomic comparisons were interpreted alongside the MLST and core-genome SNP phylogeny to contextualize potential effects of clonal relatedness.

## Results

### Prevalence of *exoU* and *exoS* genes in UTI and MK isolates

BLAST search analysis of the 23-UTI *P. aeruginosa* isolates from Bangladesh showed that 14 isolates (60.9%) carried *exoS*, whereas 9 isolates (39.1%) carried *exoU* gene (Supplementary Table 1). Among the 15 publicly available Indian UTI isolates, 9 (60.0%) were *exoU*-positive and 6 (40.0%) were *exoS*-positive (Supplementary Table 1). Among the 19 publicly available Indian MK isolates, 13 (68.4%) possessed *exoU* and 6 (31.6%) possessed *exoS* (Akter et al. 2025). No significant difference in *exoU*/*exoS* distribution was observed between Bangladeshi and Indian UTI isolates (Fisher’s exact test, *p* = 0.32) or between Indian UTI and MK isolates (*p* = 0.72). 

### Antibiotic susceptibility testing of 9 *exoU* and 14 *exoS P. aeruginosa* collected from Bangladesh

Resistance rates differed greatly between the *exoU* and *exoS* Bangladeshi UTI isolates (Table 1; Supplementary Table 2). Resistance rates of *exoU* isolates were significantly higher than *exoS* isolates for ciprofloxacin (77.8% *exoU* vs. 21.4% *exoS*,* p*_*adj*_ =0.02), levofloxacin (77.8% *exoU* vs. 21.4% *exoS*,* p*_*adj*_ =0.02) and tobramycin (77.8% *exoU* vs. 21.4% *exoS*,* p*_*adj*_ =0.02) compared with *exoS* isolates (Table 1). Although the *exoU* group had higher resistance to gentamicin (66.7% *exoU* vs. 57.1% *exoS*), this difference did not reach the statistical significance level (*p*_*adj*_ =1.00).

Among the *exoU* isolates, PA362 showed the highest ciprofloxacin MIC (1024 µg/mL), followed by PA364 (256 µg/mL). Among the *exoS* isolates, PA360 showed the highest ciprofloxacin MIC (256 µg/mL). For levofloxacin, the highest MICs among *exoU* isolates were observed in PA362 and PA364 (1024 µg/mL), followed by PA367 and PA376 (256 µg/mL). Among the *exoS* isolates, PA360 showed the highest levofloxacin MIC (128 µg/mL) (Supplementary Table 2).

High-level aminoglycoside resistance was predominantly observed among *exoU* isolates. PA362, PA364, and PA376 exhibited MICs ≥ 5120 µg/mL for both gentamicin and tobramycin, while PA357 and PA367 showed tobramycin MICs ≥ 5120 µg/mL and gentamicin MICs of 512 µg/mL. Among the *exoS* isolates, PA345 and PA348 showed the highest aminoglycoside MICs, at 512 µg/mL for both gentamicin and tobramycin.

Overall, eight isolates were resistant to all four antibiotics tested. These comprised five *exoU* isolates (PA357, PA362, PA364, PA367, and PA376) and three *exoS* isolates (PA345, PA348, and PA360).

**Table 1:** Comparison of antimicrobial susceptibility between 14 *exoS* and 9 *exoU P. aeruginosa* UTI isolates from Bangladesh

UTI Isolates from Bangladesh (*N* = 23)	*exoU* (9)		*exoS* (14)		*P*-value (raw; BH-adjusted)
	S (%)	R (%)	S (%)	R (%)	
Ciprofloxacin	22.2	77.8	78.6	21.4	0.01; 0.02
Levofloxacin	22.2	77.8	78.6	21.4	0.01; 0.02
Gentamicin	33.3	66.7	42.9	57.1	1.00; 1.00
Tobramycin	22.2	77.8	78.6	21.4	0.01; 0.02

S=Susceptible, R=Resistant

### Associations between ciprofloxacin and levofloxacin resistance and SNPs in target genes of quinolone resistance-determining region (QRDR), efflux pump genes and their regulators, and acquired resistance genes of 9 *exoU* and 14 *exoS P. aeruginosa* UTI isolates from Bangladesh

The association between ciprofloxacin and levofloxacin resistance and SNPs present in targets genes, efflux pumps and regulatory genes are presented in Table 2 and Supplementary Tables 3 and 4. Twenty-two (22) SNPs were identified with differential prevalence between *exoU* and *exoS* groups, among them several were exclusive to either *exoU* or *exoS* isolates (Table 2). The mutations detected exclusively in *exoU*-positive isolates were ParC Pro752Thr (33.33%), MexB Ser1041Glu (55.56%), MexB Val1041Ala (55.56%), MexC Ala262Glu (88.89%), Ala277Thr (66.67%), Ala378Thr (66.67%), and Glu251Gln (66.67%), MexD Thr87Ser (66.67%), MexE Ala231Thr (44.44%), Ala79Gly (44.44%), Ser8Phe (100%), MexF Ala843Thr (44.44%), NalD Val133frameshift (33.33%), OprJ Ala260Val (44.44%), OprN Ala410Ser (66.67%) (Table 2). The mutation exclusive to *exoS* isolates was MexD Glu257Gln (only found in *exoS* isolates, 50% of isolates). MexR Val126Glu and ParE Asp533Glu were each detected in 28.57% (*n* = 4) of *exoS* isolates but were more prevalent in *exoU* isolates. Some mutations were more common in *exoU* isolates than the *exoS* isolates. The OprN Ser13Pro and ParE Asp533Glu were found in all *exoU* isolates (100%), but less frequently in the *exoS* strains (21.43% and 28.57% respectively, *p*_*adj*_ < 0.01). The possession of MexC His310Arg was also prevalent in all *exoU* isolates (100%) while only 57.1% of *exoS* isolates possessed the mutation (*p*_*adj*_ = 0.05). About 77.78% of *exoU* isolates had the MexR Val126Glu mutation as compared to 28.57% of *exoS* isolates (*p*_*adj*_ = 0.04), and 55.56% of *exoU* isolates possessed the ParC Ser87Leu mutation as compared to only 7.14% of *exoS* isolates (*p*_*adj*_ = 0.02). SIFT analysis for prediction of functional mutation indicated that of the 22 identified mutations with significant differential possession, 7 were functionally disruptive mutations (Supplementary Tables 3 and 4). Out of 7 functional mutations, 5 were only present in *exoU* strains, (MexC Ala262Glu (88.89%) and Ala277Thr (66.67%); OprN Ala410Ser (66.67%); MexF Ala843Thr (44.44%); and ParC Pro752Thr (33.33%) (Table 2 and Supplementary Tables 3 and 4). The OprN Ser13Pro functional mutation was carried by 100% of *exoU* isolates, while only 21.43% of *exoS* isolates had the mutation (*p*_*adj*_ < 0.01). The Thr83Ile functional mutation in the fluoroquinolones target gene *gyrA* was present in 55.56% of *exoU* as compared to 7.14% of *exoS* isolates (*p*_*adj*_ = 0.02) (Table 2). 

Tests of associations between mutations and fluoroquinolone resistance identified strong and statistically relevant correlations which are presented in Table 2. The highest correlations were observed for GyrA Thr83Ile and ParC Ser87Leu, each exhibiting a correlation coefficient of φ = 0.68 with resistance to both ciprofloxacin and levofloxacin (*p*_*adj*_ = 0.02). Mutations in ParE Asp533Glu (φ = 0.59), MexR Val126Glu (φ = 0.56) and MexE Ser8Phe (φ = 0.55) also showed strong associations with resistance to both ciprofloxacin and levofloxacin (all *p*_*adj*_ = 0.04).

**Table 2:** Comparison of significantly different SNPs in target genes, efflux pump and regulatory genes among 9 *exoU* and 14 *exoS* UTI isolates from Bangladesh, and their associations with fluoroquinolone resistance

Gene	Mutation	*exoS*:*exoU* Frequency (%)	*P*-value (raw; BH-adjusted)	Association with ciprofloxacin resistance (φ; Fisher’s *p*-value; BH-adjusted *p*-value)	Association with levofloxacin resistance (φ; Fisher’s *p*-value; BH-adjusted *p*-value)
*gyrA*	**Thr83Ile**	7.14:55.56	0.02; 0.02	0.68; < 0.01; 0.02	0.68; < 0.01; 0.02
*parC*	**Pro752Thr**	0:33.33	0.05; 0.05	0.44; 0.07; 0.11	0.44; 0.07; 0.11
	Ser87Leu	7.14:55.56	0.02; 0.02	0.68; < 0.01; 0.02	0.68; < 0.01; 0.02
*parE*	Asp533Glu	28.57:100	< 0.01: < 0.01	0.59; 0.01; 0.04	0.59; 0.01; 0.04
*mexB*	Ser1041Glu	0:55.56	< 0.01: < 0.01	0.39; 0.13; 0.19	0.39; 0.13; 0.19
	Val1041Ala	0:55.56	< 0.01: < 0.01	0.39; 0.13; 0.19	0.39; 0.13; 0.19
*mexC*	**Ala262Glu**	0:88.89	< 0.01: < 0.01	0.46; 0.04; 0.10	0.46; 0.04; 0.10
	**Ala277Thr**	0:66.67	< 0.01: < 0.01	0.48; 0.05; 0.10	0.48; 0.05; 0.10
	Ala378Thr	0:66.67	< 0.01: < 0.01	0.48; 0.05; 0.10	0.48; 0.05; 0.10
	Glu251Gln	0:66.67	< 0.01: < 0.01	0.48; 0.05; 0.10	0.48; 0.05; 0.10
	His310Arg	57.14:100	0.05; 0.05	0.12; 0.66; 0.66	0.12; 0.66; 0.66
*mexD*	Glu257Gln	50:0	0.02; 0.02	-0.20; 0.41; 0.42	-0.20; 0.41; 0.42
	Thr87Ser	0:66.67	< 0.01: < 0.01	0.48; 0.05; 0.10	0.48; 0.05; 0.10
*oprJ*	Ala260Val	0:44.44	0.01; 0.02	0.29; 0.28; 0.32	0.29; 0.28; 0.32
*mexE*	Ala231Thr	0:44.44	0.01; 0.02	0.29; 0.28; 0.32	0.29; 0.28; 0.32
	Ala79Gly	0:44.44	0.01; 0.02	0.29; 0.28; 0.32	0.29; 0.28; 0.32
	Ser8Phe	0:100	< 0.01: < 0.01	0.55; 0.01; 0.04	0.55; 0.01; 0.04
*mexF*	**Ala843Thr**	0:44.44	0.01; 0.02	0.29; 0.28; 0.32	0.29; 0.28; 0.32
*oprN*	**Ala410Ser**	0:66.67	< 0.01: < 0.01	0.28; 0.34; 0.38	0.28; 0.34; 0.38
	**Ser13Pro**	21.43:100	< 0.01: < 0.01	0.49; 0.04; 0.10	0.49; 0.04; 0.10
*mexR*	Val126Glu	28.57:77.78	0.04; 0.04	0.56; 0.01; 0.04	0.56; 0.01; 0.04
*nalD*	Val133fs	0:33.33	0.05; 0.05	0.44; 0.07; 0.11	0.44; 0.07; 0.11

**Bold** letters denote functional mutations; *exoS: exoU* indicates the percentage of *exoS* and *exoU* isolates, respectively, carrying the indicated mutation

Eight isolates had elevated MICs for both ciprofloxacin and levofloxacin (3 *exoS* and 5 *exoU*), with MICs ranging from 16 µg/mL to 1024 µg/mL (Supplementary Table 2). Of the three *exoS* isolates (PA345, PA348 and PA360), the strongly correlated mutations GyrA Thr83Ile and ParC Ser87Leu were only observed in PA348 which showed high MICs (32 µg/mL) (Supplementary Table 3). Notably, PA360, despite lacking both mutations, displayed the highest fluoroquinolone MICs among *exoS* isolates (MIC_cip_ = 256 µg/mL, MIC_lev_ = 128 µg/mL). This isolate carried ParC Val646Leu, ParE Asp533Glu, NalC Gly71Glu and Ser209Arg, MexT Gln80fs and Phe172Ile, together with variants in the MexCD-OprJ system, including MexC Ser330Ala, MexD Ser845Ala and Glu257Gln, and OprJ Gln267Arg, Met69Val, and Ala14Val. A GyrB His148Asn mutation was observed only in PA360 *exoS* strain (Supplementary Table 3). In contrast, all 5 *exoU* isolates (PA357, PA362, PA364, PA367, and PA376) possessed both GyrA Thr83Ile and ParC Ser87Leu mutations, with MICs ranging from 128 to 1024 µg/mL. The mutations ParE Asp533Glu and MexR Val126Glu were present in nearly all high-MIC isolates, apart from PA348 which did not possess ParE Asp533Glu mutation.

The Ser13Pro functional mutation of OprN was absent only in *exoS* isolates PA348 and P360. Interestingly, PA362, which demonstrated the highest MICs (1024 µg/mL), harbored 14 SNPs across target-sites and efflux regulatory genes including multiple predicted functional and frameshift mutations (Supplementary Tables 3 and 4).

Among the high MIC isolates, a cluster consisting of PA362, PA364 and PA367 shared identical SNP profiles across target-site, efflux-associated and regulatory genes. These included functional mutations such as GyrA Thr83Ile, ParC Pro752Thr, NalC Ser209Arg and OprN Ser13Pro, together with the strongly correlated ParC Ser87Leu substitution (Supplementary Tables 3 and 4). Consistent with established QRDR mechanisms, the majority of high MIC isolates carried mutations within GyrA and/or ParC.

A limited number of mutations exclusive to resistant isolates were identified in regulatory and efflux-associated genes, including NalD Val133frame shift, NalC Glu153Gln, and MexA Lys86Glu. Several isolates also possessed unique regulatory mutations, such as multiple NalD substitutions observed exclusively in PA348 (Supplementary Table 3). SNPs that were not significantly different between 9 *exoU* and 14 *exoS* are presented in Supplementary Table 5. Interestingly, no *qnrVC* or *crpP* genes were detected in any of the 23 Bangladeshi UTI isolates. 

### Associations between aminoglycosides (gentamicin and tobramycin) resistance and SNPs in efflux pump genes and their regulators, and acquired resistance genes of 9 *exoU* and 14 *exoS P. aeruginosa* UTI isolates from Bangladesh

The identified aminoglycoside-modifying enzyme (AME) genes and mutations in efflux and regulatory genes (including *fusA2*, *parR* and *parS*) are tabulated in Table 3 and Supplementary Table 6. The phosphotransferase *aph(3′)-IIb* was detected in all 23 isolates (Supplementary Table 6) with 10 additional acquired aminoglycoside resistance genes distributed across six highly resistant isolates (MIC > 512 µg/ml; PA348, PA357, PA362, PA364, PA367, PA376). Several acquired genes were exclusive to *exoU* isolates (PA357, PA362, PA364, and PA367), including *aph(3′′)-Ib*,* aph(6)-Id*,* aac(6′)-II* and *aac(3)-Id*, whereas *rmtF* was detected only in an *exoS* isolate (PA348).

Three acquired resistance genes *aac(6′)-II*, *aac(3)-Id* and *rmtB* were identified to be differentially prevalent in *exoU* isolates (*p*_*adj*_ = 0.05) (Table 3 and Supplementary Table 6). All three were present in 33% of *exoU* isolates, but their correlation scores to gentamicin (φ = 0.31) and tobramycin (φ = 0.44) were both statistically insignificant (*p*_*adj*_ = 0.40; *p*_*adj*_ = 0.08). The acquired genes *aac(6′)-II* and *aac(3)-Id* were present on 3 *exoU* isolates (PA362, PA364 and PA367) which had the highest MIC levels to tobramycin (≥ 5120 µg/mL) and gentamicin MICs were ≥ 5120 µg/mL for PA362 and PA364 and 512 µg/mL for PA367. *rmtB* was present in PA362, PA364, and PA376, all three isolates showing MIC levels exceeding 5120 µg/mL for both gentamicin and tobramycin.

The highest levels of aminoglycoside resistance were observed in isolates PA362 and PA364, both exhibiting MICs exceeding 5120 µg/ml for gentamicin and tobramycin. These isolates harbored the greatest number of acquired AME genes (*n* = 6; *aph(3′)-IIb*,* aph(3′′)-Ib*,* aph(6)-Id*,* aac(6′)-II*,* aac(3)-Id*,* rmtB*), including *aph(3′′)-Ib* and *aph(6)-Id*, which were uniquely detected in these two isolates (Supplementary Table 6). Both also carried mutations in ParR (Leu153Arg, Ser170Asn) which were correlated to tobramycin resistance (Table 3). PA376 demonstrated comparable MIC levels and the same regulatory mutations but possessed three fewer acquired resistance genes (*aph(3′)-IIb*,* aadA11*,* rmtB*) compared to the PA362 and PA364.

In total, 16 isolates spanning both *exoU* and *exoS* genotypes demonstrated elevated aminoglycoside MICs. For gentamicin, elevated resistance (16 to ≥ 5120 µg/mL) was observed in 14 isolates (6 *exoU*, 8 *exoS*). Tobramycin resistance was more pronounced, with 10 isolates (7 *exoU*, 3 *exoS*) exhibiting elevated MICs, including six isolates with MICs ≥ 5120 µg/mL. Notably, all isolates with extreme tobramycin resistance carried the ParR Leu153Arg and Ser170Asn substitutions (Supplementary Table 6). Eight isolates (5 *exoU*, 3 *exoS*) displayed concurrent high-level resistance (MIC ≥ 512 µg/mL) to both aminoglycosides. Several mutations were observed solely in aminoglycoside-resistant isolates, including ParS Ala13Thr in *exoU* strains PA362, PA364, and PA367, and ParS Ala149Thr in PA357, all exhibiting elevated tobramycin resistance (MIC ≥ 5120 µg/mL). In FusA2, Ala197Asp was detected only in *exoU* isolates (100% vs. 0%, *p*_*adj*_ = 0.01), whereas Gln161Leu was present in 44.44% of *exoU* isolates and absent in *exoS* isolates (*p*_*adj*_ = 0.03). In correlation analysis, neither mutation showed a significant association with gentamicin resistance. For tobramycin resistance, Ala197Asp showed a significant strong correlation (φ = 0.55, *p*_*adj*_ = 0.04), while Gln161Leu showed a weak non-significant correlation (φ = 0.29, *p*_*adj*_ = 0.28). No SNPs were observed in *mexX*,* mexY* and *armZ* across the 23 isolates, with only PA356 harboring the sole OprM mutation, Ala261Thr (Supplementary Table 4). The isolate was sensitive to tobramycin but showed resistance to gentamicin (16 µg/mL).

**Table 3:** Comparison of significantly different SNPs in efflux pump and regulatory genes, together with acquired resistance genes, between 9 *exoU* and 14 *exoS P. aeruginosa* UTI isolates from Bangladesh and their association with aminoglycoside resistance

Gene	Mutation	*exoS*:*exoU* Frequency (%)	*P*-value (raw; BH-adjusted)	Association with gentamicin resistance (φ; Fisher’s *p*-value; BH-adjusted *p*-value)	Association with tobramycin resistance (φ; Fisher’s *p*-value; BH-adjusted *p*-value)
*aac(6* *′* *)-II*	-	0:33	0.05; 0.05	0.31; 0.25; 0.40	0.44; 0.07; 0.08
*aac(3)-Id*	-	0:33	0.05; 0.05	0.31; 0.25; 0.40	0.44; 0.07; 0.08
*rmtB*	-	0:33	0.05; 0.05	0.31; 0.25; 0.40	0.44; 0.07; 0.08
*fusA2*	Ala197Asp	0:100	< 0.01; 0.01	0.10; 1.00; 1.00	0.55; 0.01; 0.04
	Gln161Leu	0:44.44	0.01; 0.03	0.37; 0.13; 0.40	0.29; 0.28; 0.28
*parR*	Leu153Arg	21.43:100	< 0.01; < 0.01	-0.05; 1.00; 1.00	0.49; 0.04; 0.08
	Ser170Asn	0:100	< 0.01; < 0.01	0.10; 1.00; 1.00	0.55; 0.01; 0.04
*parS*	Ala13Thr	0:33.33	0.05; 0.05	0.31; 0.25; 0.40	0.44; 0.07; 0.08

*exoS: exoU* indicates the percentage of *exoS* and *exoU* isolates, respectively, carrying the indicated mutation or gene

### Association between 9 *exoU* and 14 *exoS* UTI isolates from Bangladesh with mutations in the DNA mismatch repair system (MMR) genes

SNPs in DNA Mismatch Repair (MMR) genes (*mutL*,* mutS*, and *uvrD*) for the 23 UTI isolates from Bangladesh are summarized in Table 4 and Supplementary Table 7. In this study, *exoU* isolates showed a median of one SNP in *mutL* (interquartile range (IQR) = 1.5–0.5), zero SNPs in *mutS* (IQR = 1 − 0), and 4 SNPs on *uvrD* IQR = 4 − 0). In comparison, the *exoS* isolates displayed a median of one SNP in *mutL* (IQR = 1 − 0), 0.5 SNPs in *mutS* (IQR = 1 − 0) and one SNP in *uvrD* (IQR = 2 − 0). Isolates with elevated MMR SNP counts (5–7 SNPs), often possessed between 32 and 40 AMR-associated SNPs. To investigate whether variation in MMR genes was associated with broader resistance-associated mutations burden, a Spearman’s Rho correlation analysis was performed. A moderate positive correlation was observed (R = 0.45, *p*_*adj*_ = 0.03) between all SNPs in *mut* genes and total AMR-associated SNP counts. Additionally, the *mutL* gene showed the strongest positive correlation (R = 0.68, *p*_*adj*_ < 0.01) with antimicrobial resistance (AMR) mutations burden. The *mutS* gene showed a moderate positive trend (R = 0.38, *p*_*adj*_ = 0.07) and the *uvrD* gene showed weak correlation (R = 0.13, *p*_*adj*_ = 0.55), with both failing to reach statistical significance.

**Table 4:** SNPs in the DNA mismatch repair (MMR) system genes of 9 *exoU* and 14 *exoS* UTI isolates from Bangladesh

Strain ID	T3SS	*mutL*	*mutS*	*uvrD*	Total Number of SNPs in *mut* genes^1^	Total number of SNPs in AMR-associated genes^2^
PA331	*exoS*	1	1	0	2	58
PA333	*exoS*	0	0	0	0	11
PA334	*exoS*	0	0	4	4	15
PA335	*exoS*	0	0	0	0	11
PA341	*exoS*	1	1	0	2	58
PA345	*exoS*	1	1	0	2	58
PA348	*exoS*	0	3	1	4	29
PA356	*exoS*	1	0	0	1	14
PA360	*exoS*	0	0	2	2	20
PA368	*exoS*	0	1	1	2	18
PA371	*exoS*	0	0	2	2	16
PA373	*exoS*	0	0	2	2	17
PA374	*exoS*	0	1	2	3	23
PA375	*exoS*	0	1	2	3	23
PA338	*exoU*	2	1	4	7	32
PA343	*exoU*	1	0	0	1	25
PA353	*exoU*	2	1	4	7	32
PA357	*exoU*	1	1	0	2	34
PA362	*exoU*	1	0	4	5	40
PA364	*exoU*	1	0	4	5	40
PA367	*exoU*	1	0	4	5	40
PA372	*exoU*	1	0	4	5	36
PA376	*exoU*	1	0	0	1	27

^1^*mut* genes included *mutL*,* mutS*, and *uvrD*. ^2^AMR-related genes comprised *gyrA*,* gyrB*,* parC*,* parE*,* nfxB*,* mexR*,* nalC*,* nalD*,* mexT*,* mexA*,* mexB*,* oprM*,* mexC*,* mexD*,* oprJ*,* mexE*,* mexF*,* oprN*,* parR*,* parS*, and *fusA2*

### Comparison of different SNPs in target genes of QRDR, efflux pump and regulatory genes of 9 *exoU* and 6 *exoS P. aeruginosa* UTI isolates from India

The SNPs that were differentially prevalent between 9 *exoU* and 6 *exoS P. aeruginosa* UTI isolates across target site genes, efflux systems and their regulatory genes of fluoroquinolone resistance are tabulated in Table 5 and functional mutations are presented in Supplementary Tables 8 and 9.

Overall, *exoU* isolates possessed a higher number of SNPs in the target site (QRDR) and efflux pump regulatory genes than the *exoS* isolates in the Indian UTI cohort. The functional mutation GyrA Thr83Ile was more prevalent in *exoU* (100%) isolates than *exoS* (33%, *p*_*adj*_= 0.02) (Table 5). A similar prevalence was observed for ParE Asp533Glu as 100% of *exoU* and 17% of *exoS* isolates had the mutation (*p*_*adj*_ = 0.01). For ParC, the mutation Val646Leu was exclusive to *exoS* isolates, being present in 50% of those isolates (*p*_*adj*_ = 0.04). The only GyrB mutation, Glu468Asp was identified on strain U804 (Supplementary Table 8).

On efflux pump genes, the Ser1041Glu and Val1041Ala mutations in MexB were detected only in *exoU* isolates (78%, *p*_*adj*_ = 0.02) (Table 5 and Supplementary Table 9). The MexC Ala262Glu functional mutation was also exclusive to *exoU* isolates (89%, *p*_*adj*_ = 0.01). The other mutation on MexC, Pro383Ser was identified on all *exoU* isolates as compared to 17% of *exoS* isolates (*p*_*adj*_ = 0.01). Two mutations in MexD, Val434Ala and Lys1031Arg, were detected in 56% of *exoU* isolates, while neither mutation was found in any *exoS* isolate (*p*_*adj*_ = 0.04). On OprJ, the Met69Val mutation was only present in *exoS* isolates (67%), while the functional mutation OprJ Thr25Ile was solely found in *exoU* isolates at 56%. The MexE mutation Ser8Phe was identified in 17% of *exoS* and 89% of *exoU* isolates (*p*_*adj*_ = 0.02), while the OprN mutation Ser13Pro was present only in all *exoU* isolates (*p*_*adj*_= 0.01).

**Table 5:** Comparison of significantly different SNPs in target genes, efflux pump and regulatory genes among 9 *exoU* and 6 *exoS* UTI Indian isolates

Gene	Mutation	*exoS*:*exoU* Frequency (%)	*P*-value (raw; BH-adjusted)
***gyrA***	**Thr83Ile**	33:100	0.01; 0.02
***parC***	Val646Leu	50:0	0.04; 0.04
***parE***	Asp533Glu	17:100	< 0.01; 0.01
***mexB***	Ser1041Glu	0:78	< 0.01; 0.02
	Val1041Ala	0:78	< 0.01; 0.02
***mexC***	**Ala262Glu**	0:89	< 0.01; 0.01
	Pro383Ser	17:100	< 0.01; 0.01
***mexD***	Lys1031Arg	0:56	0.04; 0.04
	Val434Ala	0:56	0.04; 0.04
***oprJ***	Met69Val	67:0	0.01; 0.02
	**Thr25Ile**	0:56	0.04; 0.04
***mexE***	Ser8Phe	17:89	0.01; 0.02
***oprN***	**Ser13Pro**	0:100	< 0.01; 0.01
***mexR***	Val126Glu	17:100	< 0.01; 0.01
***nalC***	**Gly213Asp**	0:56	0.04; 0.04
	**Ser209Arg**	50:100	0.04; 0.04
	Ser5fs	0:56	0.04; 0.04

**Bold** letters denote functional mutations; *exoS: exoU* indicates the percentage of *exoS* and *exoU* isolates, respectively, carrying the indicated mutation

For MexR, Val126Glu was present on all *exoU* isolates where less than one fifth (17%) of *exoS* isolates possessed that mutation (*p*_*adj*_ = 0.01). Three NalC mutations were observed to be differentially prevalent, of which Ser5 frame shift and functional mutation Gly213Asp was present in only *exoU* isolates (56%, *p*_*adj*_ = 0.04). whilst functional mutation Ser209Arg were identified in 100% of *exoU* isolates and half of the *exoS* isolates (*p*_*adj*_ = 0.04). Mutations which were not significantly different between 9 *exoU* and 6 *exoS* Indian UTI isolates are presented in Supplementary Table 10. 

### Comparison of different acquired aminoglycosides resistance genes, SNPs in efflux pump and regulatory genes of 9 *exoU* and 6 *exoS* UTI isolates from India

The presence of aminoglycoside resistance genes and the mutations associated with their regulatory components that were differentially prevalent between 9 *exoU* and 6 *exoS P. aeruginosa* UTI isolates from India are presented in Table 6 and the distribution of acquired resistance genes is showed in Supplementary Table 11. Analysis of acquired aminoglycoside resistance genes revealed distinct distribution patterns between *exoU* and *exoS* isolates. The phosphotransferase gene *aph(3′)-IIb* was present in all isolates from both groups, indicating its conserved presence across two groups. Several acquired resistance genes were more frequently detected in *exoU* isolates. Notably, *aph(3′′)-Ib*,* aph(6)-Id*, and *rmtF* were each identified in 66.7% (6/9) of *exoU* isolates, whereas their prevalence in *exoS* isolates was 16.7% (1/6), 16.7% (1/6), and 0% (0/6), respectively (Supplementary Table 11). Among these, only *rmtF* was significantly more prevalent in the *exoU* group than in the *exoS* group (66.7% vs. 0%, *p*_*adj*_ = 0.04), whereas the higher prevalence of *aph(3′′)-Ib* and *aph(6)-Id* in *exoU* isolates did not reach statistical significance. Additional acquired resistance genes, including *aac(6′)-II*,* aac(3)-Id*,* ant(2′′)-Ia*,* rmtD2*, and *rmtB*, were detected exclusively or predominantly in *exoU* isolates at low frequencies (11.1–22.2%) and were not observed in *exoS* isolates (Supplementary Table 11). In contrast, *aph(3′)-VIa* was detected only in *exoS* isolates (16.7%), while *aadA11* was observed in both groups at low frequencies.

The *exoU* isolates were found to harbor larger burden of SNPs in *fusA2* and *parR* genes compared to the *exoS* isolates (Supplementary Table 11). In FusA2, Gln161Leu were prevalent in only *exoU* group (67%, *p*_*adj*_ = 0.04). In case of ParR, Leu153Arg and Ser170Asn were predominantly associated with *exoU* being present in 100% isolates compared to only 17% *exoS* isolates (*p*_*adj*_ <0.01) (Table 6). No SNPs in *parS* gene were significantly different between the two genotype groups (Supplementary Table 11).

**Table 6:** Comparison of significantly different SNPs in efflux pump and regulatory genes together with acquired resistance genes associated with aminoglycosides resistance between 9 *exoU* and 6 *exoS P. aeruginosa* UTI isolates from India

Gene	Mutation	*exoS*:*exoU* Frequency (%)	*P*-value (raw; BH-adjusted)
*rmtF*	-	0:67	0.03; 0.04
*fusA2*	Gln161Leu	0:67	0.03; 0.04
*parR*	Leu153Arg	17:100	< 0.01; < 0.01
	Ser170Asn	17:100	< 0.01; < 0.01

*exoS: exoU* indicates the percentage of *exoS* and *exoU* isolates, respectively, carrying the indicated mutation or gene

### Association of 9 *exoU* and 6 *exoS* UTI isolates from India and mutations in the DNA mismatch repair system (MMR) genes

The SNPs in the DNA Mismatch Repair (MMR) genes (*mutL*,* mutS* and *uvrD*) are shown in Table 7 and Supplementary Table 12. Out of the 9 *exoU* isolates, 7 contained a total of 7 SNPs in these genes. The *exoS* isolates contained a maximum of four MMR SNPs with an average of two MMR mutations across the 6 *exoS* isolates. Analyzing the number of SNPs, *exoU* isolate showed a median of two SNPs on *mutL* gene (IQR:2 − 1), one SNP in *mutS* gene (IQR:1 − 0) and four SNPs in *uvrD* gene (IQR:4 – 4). In contrast, the *exoS* isolates showed a median of zero SNPs in *mutL* gene (IQR:1 − 0), one SNP in *mutS* gene (IQR:1 − 0) and 0.5 mutations in *uvrD* gene (IQR:1 − 0). Additionally, the *exoU* isolates with 5–7 SNPs were found to contain 34–42 AMR associated SNPs. A correlation analysis between total MMR SNP counts and total AMR-associated SNP counts showed a strong positive correlation (R = 0.74, *p*_*adj*_ < 0.01). The *uvrD* (R = 0.76, *p*_*adj*_ < 0.01) and *mutL* (R = 0.65, *p*_*adj*_ = 0.02) demonstrated strong statistically significant positive correlations to AMR mutations burden. Whilst *mutS* showed no correlation (*R* = 0, *p*_*adj*_ = 1).

**Table 7:** SNPs in the DNA mismatch repair (MMR) system genes of 9 *exoU* and 6 *exoS* UTI isolates from India

Strain ID	T3SS	*mutL*	*mutS*	*uvrD*	Total Number of SNPs in *mut* genes^1^	Total number of SNPs in AMR-associated genes^2^
AI_1323	*exoS*	0	0	4	4	19
AI_1678	*exoS*	0	1	1	2	16
AI_3297	*exoS*	1	1	0	2	19
AI_3300	*exoS*	1	1	0	2	19
PA_2703	*exoS*	0	0	1	1	26
U804	*exoS*	0	1	0	1	14
AI_1294	*exoU*	2	0	4	6	37
AI_1609	*exoU*	1	0	4	5	42
MGL_146_B9	*exoU*	2	1	4	7	38
MGL_95_B5	*exoU*	2	1	4	7	41
*P.aeruginosa*-32	*exoU*	2	1	4	7	38
*P.aeruginosa*-33	*exoU*	2	1	4	7	38
*P.aeruginosa*-36	*exoU*	2	1	4	7	38
PA790	*exoU*	2	1	4	7	34
VRFPA08	*exoU*	1	1	5	7	39

^1^*mut* genes included *mutL*,* mutS*, and *uvrD*. ^2^AMR-related genes comprised of *gyrA*,* gyrB*,* parC*,* parE*,* nfxB*,* mexR*,* nalC*,* nalD*,* mexT*,* mexA*,* mexB*,* oprM*,* mexC*, *oprJ*, *mexE*,* mexF*,* oprN*,* parR*,* parS*, and *fusA2*

### Differences in the SNPs present in target genes, efflux pump and regulatory genes of fluoroquinolone and aminoglycosides resistance between Bangladeshi and Indian UTI isolates

The SNPs in target genes, efflux pump and regulatory genes of fluoroquinolone and aminoglycosides resistance in UTI isolates of India and Bangladesh are presented in Table 8. A comparison of mutation frequencies between *P. aeruginosa* UTI isolates from Bangladesh and India revealed a consistent enrichment of resistance-associated mutations in *exoU* strains compared with *exoS* strains across both cohorts, although the magnitude of differences varied. Overall, differentially prevalent SNPs were predominantly either more frequent or exclusive to *exoU* isolates in both countries (Table 8).

Of the 20 mutations identified as differentially prevalent in Indian isolates, 18 mutations were either exclusively on *exoU* isolates or more commonly present in *exoU* isolates. The remaining 2 mutations ParC Val646Leu and OprJ Met69Val were present only in *exoS* isolates. Similarly, among the 27 mutations identified as differentially possessed by Bangladesh UTI isolates, only MexD (Glu257Gln) was detected in *exoS* isolates, while the remaining 26 mutations were observed either solely or at higher frequencies in *exoU* isolates.

In both datasets, several mutations showed similar distributions according to *exoU/exoS* status in both Bangladeshi and Indian UTI isolates, with GyrA (Thr83Ile), ParE (Asp533Glu), MexB (Ser1041Glu and Val1041Ala), MexC (Ala262Glu), MexE (Ser8Phe), OprN (Ser13Pro), MexR (Val126Glu), FusA2 (Gln161Leu), and ParR (Leu153Arg and Ser170Asn) occurring more frequently in *exoU* than in *exoS* isolates in both datasets (Table 8). In contrast, MexD (Glu257Gln) in Bangladeshi isolates and ParC (Val646Leu) and OprJ (Met69Val) in Indian isolates were detected only in *exoS* strains.

Despite these overlapping patterns, additional differences were evident between the two UTI populations. In the Bangladeshi isolates, mutations that were more prevalent in the *exoU* group included ParC (Pro752Thr and Ser87Leu), MexC (Ala277Thr, Ala378Thr, Glu251Gln, and His310Arg), MexD (Thr87Ser), OprJ (Ala260Val), MexE (Ala231Thr and Ala79Gly), MexF (Ala843Thr), OprN (Ala410Ser), NalD (Val133fs), FusA2 (Ala197Asp), and ParS (Ala13Thr). In addition, the acquired aminoglycoside resistance genes *aac(6′)-II* and *aac(3)-Id* were found only in Bangladeshi *exoU* isolates.

By comparison, Indian UTI isolates were characterized by a different set of *exoU*-associated mutations, including MexC (Pro383Ser), MexD (Lys1031Arg and Val434Ala), OprJ (Thr25Ile), and NalC variants (Gly213Asp, Ser209Arg, and Ser5fs). The acquired aminoglycoside resistance determinant *rmtF* was significantly more prevalent in *exoU* isolates only in the Indian UTI dataset.

**Table 8:** Comparison of differentially prevalent SNPs of QRDR, efflux pumps and their regulatory genes, possession of acquired resistance genes between Bangladeshi UTI (9 *exoU* and 14 *exoS*) and Indian UTI (9 *exoU* and 6 *exoS*) isolates

Gene	Mutation	*exoS*:*exoU* Frequency (%) in Bangladeshi UTI	*P*-value (raw; BH-adjusted)	*exoS*:*exoU* Frequency (%) in Indian UTI	*P*-value (raw; BH-adjusted)
*gyrA*	**Thr83Ile**	7.14:55.56	0.02; 0.02	33:100	0.01; 0.02
*parC*	**Pro752Thr**	0:33.33	0.05; 0.05		
	Ser87Leu	7.14:55.56	0.02; 0.02		
	Val646Leu			50:0	0.04; 0.04
*parE*	Asp533Glu	28.57:100	< 0.01; < 0.01	17:100	< 0.01; 0.01
*mexB*	Ser1041Glu	0:55.56	< 0.01; < 0.01	0:78	< 0.01; 0.02
	Val1041Ala	0:55.56	< 0.01; < 0.01	0:78	< 0.01; 0.02
*mexC*	**Ala262Glu**	0:88.89	< 0.01; < 0.01	0:89	< 0.01; 0.01
	**Ala277Thr**	0:66.67	< 0.01; < 0.01		
	Ala378Thr	0:66.67	< 0.01; < 0.01		
	Glu251Gln	0:66.67	< 0.01; < 0.01		
	His310Arg	57.14:100	0.05; 0.05		
	Pro383Ser			17:100	< 0.01; 0.01
*mexD*	Glu257Gln	50:0	0.02; 0.02		
	Thr87Ser	0:66.67	< 0.01; < 0.01		
	Lys1031Arg			0:56	0.04; 0.04
	Val434Ala			0:56	0.04; 0.04
*oprJ*	Ala260Val	0:44.44	0.01; 0.02		
	Met69Val			67:0	0.01; 0.02
	**Thr25Ile**			0:56	0.04; 0.04
*mexE*	Ala231Thr	0:44.44	0.01; 0.02		
	Ala79Gly	0:44.44	0.01; 0.02		
	Ser8Phe	0:100	< 0.01; < 0.01	17:89	0.01; 0.02
*mexF*	**Ala843Thr**	0:44.44	0.01; 0.02		
*oprN*	**Ala410Ser**	0:66.67	< 0.01; < 0.01		
	**Ser13Pro**	21.43:100	< 0.01; < 0.01	0:100	< 0.01; 0.01
*mexR*	Val126Glu	28.57:77.78	0.04; 0.04	17:100	< 0.01; 0.01
*nalC*	**Gly213Asp**			0:56	0.04; 0.04
	**Ser209Arg**			50:100	0.04; 0.04
	Ser5fs			0:56	0.04; 0.04
*nalD*	Val133fs	0:33.33	0.05; 0.05		
*fusA2*	Ala197Asp	0:100	< 0.01; 0.01		
	Gln161Leu	0:44.44	0.01; 0.03	0:67	0.03; 0.04
*parR*	Leu153Arg	21.43:100	< 0.01; < 0.01	17:100	< 0.01; < 0.01
	Ser170Asn	0:100	< 0.01; < 0.01	17:100	< 0.01; < 0.01
*parS*	Ala13Thr	0:33.33	0.05; 0.05		
*aac(6’)-II*	-	0:33	0.05; 0.05		
*aac(3)-Id*	-	0:33	0.05; 0.05		
*rmtB*	-	0:33	0.05; 0.05		
*rmtF*	-			0:67	0.03; 0.04

**Bold** font indicates functional mutations; *exoS: exoU* indicates the percentage of *exoS* and *exoU* isolates, respectively, carrying the indicated mutation or gene

### Comparison of DNA mismatch repair gene mutations in Bangladeshi and Indian *exoU* and *exoS* isolates and their association with SNPs in AMR-associated genes

Among the MMR genes examined, only *mutL* differed significantly between *exoU* and *exoS* isolates, and this was observed only in the Bangladeshi collection, where it was more frequent in *exoU* isolates (77.78%) than in *exoS* isolates (28.57%) (*p*_*adj*_ = 0.04) (Table 9). No significant differences were found for *mutS* or *uvrD* in either Bangladeshi or Indian isolates. In correlation analysis, *mutL* was positively associated with the total number of SNPs in AMR-associated genes in both Bangladeshi (R = 0.68, *p*_*adj*_ < 0.01) and Indian isolates (R = 0.65, *p*_*adj*_ = 0.02), whereas *uvrD* showed a significant correlation only in Indian isolates (R = 0.76, *p*_*adj*_ < 0.01). No significant correlation was observed for *mutS* in either population.

**Table 9:** Differences in SNPs possession in the DNA mismatch repair (MMR) system genes between *exoU* and *exoS* UTI isolates from Bangladesh and India and association with SNPs in AMR-associated genes

Country	Frequency of SNP possession (%) – *exoS:exoU*	Difference between *exoS* and *exoU* (raw *p*; BH-adjusted *p*)	Gene	Median SNP		Interquartile Range (IQR)		Correlation of MMR genes with total number of SNPs in AMR-associated genes^1^ (R, raw *p*; BH-adjusted *p*)
				*exoU*	*exoS*	*exoU*	*exoS*	
Bangladesh	28.57:77.78	0.04; 0.04	*mutL*	1	1	1.5–0.5	1 − 0	0.68; < 0.01; < 0.01
	50:33.33	0.67; 1.00	*mutS*	0	0.5	1 − 0	1 − 0	0.38; 0.07; 0.10
	57.14:66.67	1.00; 1.00	*uvrD*	4	1	4 − 0	2 − 0	0.13; 0.55; 0.55
India	33.33:88.89	0.09; 0.27	*mutL*	2	0	2 − 1	1 − 0	0.65; < 0.01; 0.02
	66.67:66.67	1.00; 1.00	*mutS*	1	1	1 − 0	1 − 0	0; 1.00; 1.00
	50:88.89	0.24; 0.35	*uvrD*	4	0.5	4 – 4	1 − 0	0.76; < 0.01; < 0.01

*exoS: exoU* represents the percentage of *exoS* and *exoU* isolates, respectively, in which SNPs were detected in the indicated MMR gene. ^1^AMR-related genes comprised of *gyrA*,* gyrB*,* parC*,* parE*,* nfxB*,* mexR*,* nalC*,* nalD*,* mexT*,* mexA*,* mexB*,* oprM*,* mexC*, *oprJ*, *mexE*,* mexF*,* oprN*,* parR*,* parS*, and *fusA2*

### Differences in the SNPs present in target genes, efflux pump and regulatory genes of fluoroquinolone and aminoglycosides resistance between Indian MK and UTI isolates

Comparative analysis of SNPs in Indian keratitis and Indian UTI isolates across target genes, efflux pump and regulatory genes of fluoroquinolone and aminoglycosides resistance revealed both shared and infection-specific mutation patterns between *exoU* and *exoS* isolates (Table 10). A core set of mutations was consistently enriched in *exoU* isolates across both infection types. These included key QRDR mutations such as GyrA (Thr83Ile) and ParE (Asp533Glu), together with efflux-associated variants including MexR (Val126Glu), MexB (Ser1041Glu), and MexC (Ala262Glu), all of which showed significantly higher prevalence in *exoU* compared with *exoS* isolates in both keratitis and UTI datasets (*p*_*adj*_ ≤ 0.04). In contrast, OprJ (Met69Val) showed an inverse distribution and was detected only in *exoS* isolates in both infection types.

Among Indian keratitis isolates, additional mutations showed significantly different prevalence between the *exoU* and *exoS* isolates, including ParC (Ser87Ile), NalC (Asp79Glu), MexS (Val73Ala), MexB (Val1042Ala), MexC (Glu251Gln and Ala277Thr), MexD (Thr87Ser), MexX (Ala30Thr), and MexY (Ile536Val), with these mutations being enriched in *exoU* isolates. In contrast, FusA2 Gln161Leu was identified only in UTI isolates and was detected in 67% of *exoU* isolates, while remaining absent in *exoS* isolates (*p*_*adj*_ = 0.04). The acquired resistance determinant *crpP* was detected only in keratitis isolates, although the difference between two groups was not statistically significant. In contrast, Indian UTI isolates had distinct sets of additional mutations that were significantly more prevalent in the *exoU* than the *exoS* isolates. These were NalC variants (Gly213Asp, Ser209Arg, and Ser5fs); MexD (Lys1031Arg and Val434Ala); MexE (Ser8Phe); OprJ (Thr25Ile); OprN (Ser13Pro); and ParR (Leu153Arg and Ser170Asn). Noticeably, ParC (Val646Leu) was significantly more common in *exoS* than *exoU* UTI isolates. The acquired resistance determinant *qnrVC1* was identified in both keratitis and UTI isolates, but its prevalence did not differ significantly between the groups in either dataset, whereas *rmtF* was detected only in the UTI dataset and showed a significant association with *exoU* isolates.

**Table 10:** Comparison of differentially prevalent SNPs in QRDR, efflux pumps and their regulatory genes, possession of acquired resistance genes associated with fluoroquinolones and aminoglycosides between Indian keratitis (13 *exoU* and 6 *exoS)* and UTI (9 *exoU* and 6 *exoS*) isolates

Gene	Mutation	Indian keratitis isolates		Indian UTI isolates	
		Frequency of possession (%)-*exoS*:*exoU*	Difference between *exoS* and *exoU* (raw *p*; BH-adjusted *p*)	Frequency of possession (%)-*exoS*:*exoU*	Difference between *exoS* and *exoU* (raw *p*; BH-adjusted *p*)
*gyrA*	**Thr83Ile**	0:100	< 0.01; < 0.01	33:100	0.01; 0.02
*parC*	Ser87Ile	0:100	< 0.01; < 0.01		
	Val646Leu			50:0	0.04; 0.04
*parE*	Asp533Glu	16.7:100	< 0.01; < 0.01	17:100	< 0.01; 0.01
*mexR*	Val126Glu	50:100	0.02; 0.03	17:100	< 0.01; 0.01
*nalC*	Asp79Glu	0:92.3	< 0.01; < 0.01		
	**Gly213Asp**			0:56	0.04; 0.04
	**Ser209Arg**			50:100	0.04; 0.04
	Ser5fs			0:56	0.04; 0.04
*mexS*	Val73Ala	0:53.8	0.04; 0.04		
*mexB*	Ser1041Glu	0:84.6	< 0.01; < 0.01	0:78	< 0.01; 0.02
	Val1042Ala	0:84.6	< 0.01; < 0.01		
	Val1041Ala			0:78	< 0.01; 0.02
*mexC*	Glu251Gln	0:53.8	0.04; 0.04		
	**Ala262Glu**	0:61.5	0.02; 0.03	0:89	< 0.01; 0.01
	**Ala277Thr**	0:53.8	0.04; 0.04		
	Pro383Ser			17:100	< 0.01; 0.01
*mexD*	Thr87Ser	0:53.8	0.04; 0.04		
	Lys1031Arg			0:56	0.04; 0.04
	Val434Ala			0:56	0.04; 0.04
*mexE*	Ser8Phe			17:89	0.01; 0.02
*oprJ*	Met69Val	50:0	0.02; 0.03	67:0	0.01; 0.02
	**Thr25Ile**			0:56	0.04; 0.04
*oprN*	**Ser13Pro**			0:100	< 0.01; 0.01
*mexX*	Ala30Thr	16.7:100	< 0.01; < 0.01		
*mexY*	Ile536Val	0:84.6	< 0.01; < 0.01		
*fusA2*	Gln161Leu			0:67	0.03; 0.04
*parR*	Leu153Arg			17:100	< 0.01; < 0.01
	Ser170Asn			17:100	< 0.01; < 0.01
*crpP*	-	50:92.3	0.07; 0.08		
*qnrVC1*	-	0:33.3	0.26; 0.26	0:22.22	0.48; 0.49
*rmtF*	-			0:67	0.03; 0.04

**Bold** font indicates functional mutations; *exoS: exoU* indicates the percentage of *exoS* and *exoU* isolates, respectively, carrying the indicated mutation or gene

### Comparison of DNA mismatch repair gene SNPs between *exoU *and *exoS *in Indian MK and UTI isolates and their association with SNPs in AMR-associated genes

In the Indian isolates, *mutL* and *uvrD* showed significant differences between *exoU* and *exoS* groups in keratitis isolates (*p*_*adj*_ < 0.01), whereas no significant differences were observed in UTI isolates (Table 11). Correlation analysis revealed that *mutL* was positively associated with SNPs in AMR-associated genes in both keratitis (R = 0.57, *p*_*adj*_ = 0.04) and UTI isolates (R = 0.65, *p*_*adj*_ = 0.02), while *uvrD* showed a significant correlation only in UTI isolates (R = 0.76, *p*_*adj*_ < 0.01). No significant associations were observed for *mutS* in either group.

**Table 11:** Differences in SNPs possession in the DNA mismatch repair (MMR) system genes between 13 *exoU* and 6 *exoS* Indian keratitis and 9 *exoU* and 6 *exoS* Indian UTI isolates and association with SNPs in AMR-associated genes

Sample Type	Frequency of SNP possession (%) – *exoS:exoU*	Difference between *exoS* and *exoU* (raw *p*; BH-adjusted *p*)	Gene	Median SNP		Interquartile Range (IQR)		Correlation of MMR genes with total number of SNPs in AMR-associated genes^1^ (R, raw *p*; BH-adjusted *p*)
				*exoU*	*exoS*	*exoU*	*exoS*	
Keratitis	0:84.6	< 0.01; < 0.01	*mutL*	1	0	0	0	0.57; 0.01; 0.04
	16.7:53.9	0.18; 0.18	*mutS*	1	0	1 − 0	0	0.13; 0.59; 0.59
	3.3:100	< 0.01; < 0.01	*uvrD*	4	0	5 − 4	2 − 0	0.31; 0.20; 0.37
UTI	33.33:88.89	0.09; 0.27	*mutL*	2	0	2 − 1	1 − 0	0.65; < 0.01; 0.02
	66.67:66.67	1.00; 1.00	*mutS*	1	1	1 − 0	1 − 0	0; 1.00; 1.00
	50:88.89	0.24; 0.35	*uvrD*	4	0.5	4 – 4	1 − 0	0.76; < 0.01; < 0.01

*exoS: exoU* represents the percentage of *exoS* and *exoU* isolates, respectively, in which SNPs were detected in the indicated MMR gene. ^1^AMR-related genes comprised of *gyrA*,* gyrB*,* parC*,* parE*,* nfxB*,* mexR*,* nalC*,* nalD*,* mexT*,* mexA*,* mexB*,* oprM*,* mexC*, *oprJ*, *mexE*,* mexF*,* oprN*,* parR*,* parS*, and *fusA2*

### Distribution of shared and cohort-specific SNPs across Bangladesh UTI, Indian UTI, and Indian MK cohorts

**Fig. 1:**
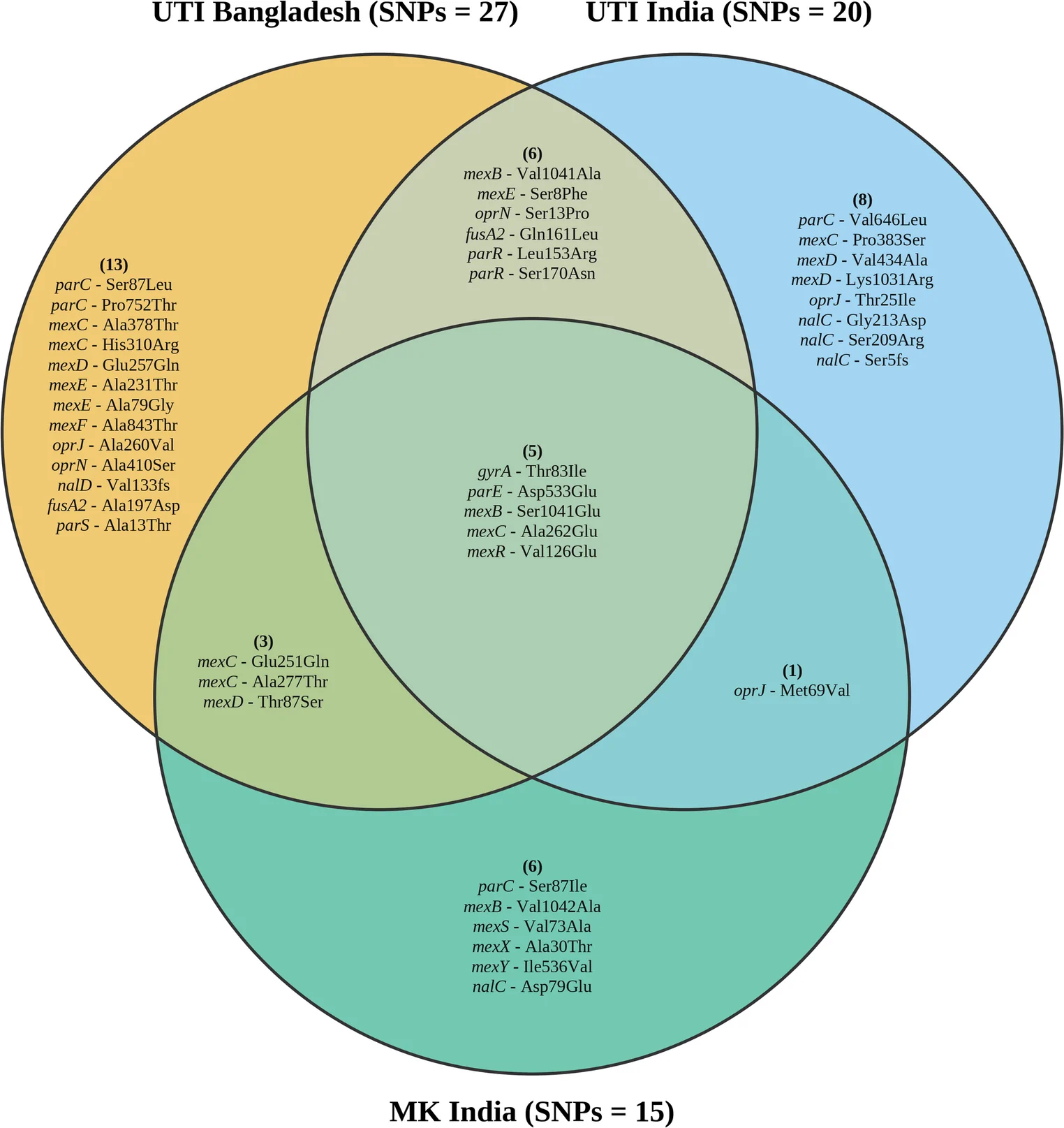
Venn diagram showing shared and cohort-specific SNPs with significantly different prevalence between *exoU*- and *exoS*-positive *P. aeruginosa* isolates from the Bangladesh UTI, Indian UTI, and Indian MK cohorts. Numbers within each section indicate the number of SNPs unique to or shared among the respective cohorts

A Venn diagram was constructed to compare mutations showing significantly different prevalence between *exoU*- and *exoS*-positive *P. aeruginosa* isolates across the Bangladesh UTI, Indian UTI, and Indian MK cohorts (Fig. 1). A total of 27 such SNPs were identified in the Bangladesh UTI cohort, 20 in the Indian UTI cohort, and 15 in the Indian MK cohort. Five mutations were shared across all three cohorts: GyrA Thr83Ile, ParE Asp533Glu, MexB Ser1041Glu, MexC Ala262Glu, and MexR Val126Glu.

Six mutations were shared exclusively between the Bangladesh and Indian UTI cohorts: MexB Val1041Ala, MexE Ser8Phe, OprN Ser13Pro, FusA2 Gln161Leu, and ParR Leu153Arg and Ser170Asn. Three SNPs, MexC Glu251Gln, MexC Ala277Thr, and MexD Thr87Ser, were shared between the Bangladesh UTI and Indian MK cohorts, whereas OprJ Met69Val was the only SNP shared exclusively between the Indian UTI and Indian MK cohorts. Thirteen SNPs were unique to the Bangladesh UTI cohort, eight to the Indian UTI cohort, and six to the Indian MK cohort. These findings demonstrate both shared and cohort-specific patterns of mutations differentially prevalent between *exoU*- and *exoS*-positive isolates. 

### Comparison of virulence factors genes between *exoU* and *exoS P. aeruginosa* UTI isolates from Bangladesh and India

VFDB compared 17 virulence factors classes encompassing 327 virulence-associated genes of 9 *exoU* and 14 *exoS P. aeruginosa* Bangladeshi UTI isolates and identified significant differences across adherence, enzyme, and iron uptake classes between *exoU* and *exoS* (Table 12 and Supplementary Table 13). Four type IV pili biosynthesis genes - *pilC* (64.3% vs. 11.1%; *p*_*adj*_ = 0.03), *pilV* (71.4% vs. 22.2%; *p*_*adj*_ = 0.04), *pilW* (85.7% vs. 22.2%; *p*_*adj*_ = 0.01), and *pilY2* (71.4% vs. 11.1%; *p*_*adj*_ = 0.01) were significantly enriched in *exoS* isolates as compared to *exoU*. Conversely, *pldA* was detected in all *exoU* isolates (100%) compared to only 35.7% of *exoS* isolates (*p*_*adj*_ = 0.01), and the pyoverdine biosynthesis gene *pvdY* was exclusively present in *exoU* strains (100% vs. 0%; *p*_*adj*_ < 0. 01).

**Table 12:** Differences of virulence factors genes between 9 *exoU* and 14 *exoS P. aeruginosa* UTI isolates from Bangladesh

Virulence genes	*exoU* (%)	*exoS* (%)	*P*-value (raw; BH-adjusted)
*pilC*	11.1	64.3	0.03; 0.03
*pilV*	22.2	71.4	0.04; 0.04
*pilW*	22.2	85.7	0.01; 0.01
*pilY2*	11.1	71.4	0.01; 0.01
*pldA*	100	35.7	< 0.01; 0.01
*pvdY*	100	00.0	< 0.01; <0.01

In the Indian cohort, VFDB analysis of 9 *exoU* and 6 *exoS P. aeruginosa* isolates identified significant differences in two genes across two different virulence factors classes (adherence and regulation) (Table 13 and Supplementary Table 14). The type IV pili biosynthesis gene *pilC* was detected in 50% of *exoS* isolates but was completely absent in *exoU* isolates (*p*_*adj*_ = 0.04). Conversely, the regulatory gene *csrA* was only present in *exoU* strains (55.6% vs. 0%; *p*_*adj*_ = 0.04).

**Table 13:** Differences of virulence factors genes between 9 *exoU* and 6 *exoS P. aeruginosa* UTI isolates from India

Virulence genes	*exoU* (%)	*exoS* (%)	*P*-value (raw; BH-adjusted)
*pilC*	0	50	0.04; 0.04
*csrA*	55.6	0	0.04; 0.04

### Comparison of virulence factor genes between *exoU* and *exoS P. aeruginosa* from Indian MK and UTI isolates

Comparison of virulence factor gene distributions between the Indian UTI cohort (9 *exoU* and 6 *exoS*) and the Indian MK cohort (13 *exoU* and 6 *exoS*) from previously published dataset (Akter et al. 2026d), revealed infection-specific differences. In Indian MK, *pldA* gene was more prevalent in *exoU* than *exoS* isolates (92.3% vs. 33.3%; *p*_*adj*_ = 0.05) and *pilA* gene was significantly enriched in *exoS* isolates (50% vs. 0%; *p*_*adj*_ = 0.05), a pattern not observed in UTI cohort. In contrast, In Indian UTI, *csrA* was detected only in *exoU* isolates (55.6% vs. 0%; *p*_*adj*_ = 0.04) while *pilC* gene was significantly more common in *exoS* isolates (50% vs. 0%; *p*_*adj*_ = 0.04).

### Multilocus sequence typing, core-genome SNP phylogenetic analysis, and clonal context of resistance, virulence, and MMR-gene variation

**Fig. 2:**
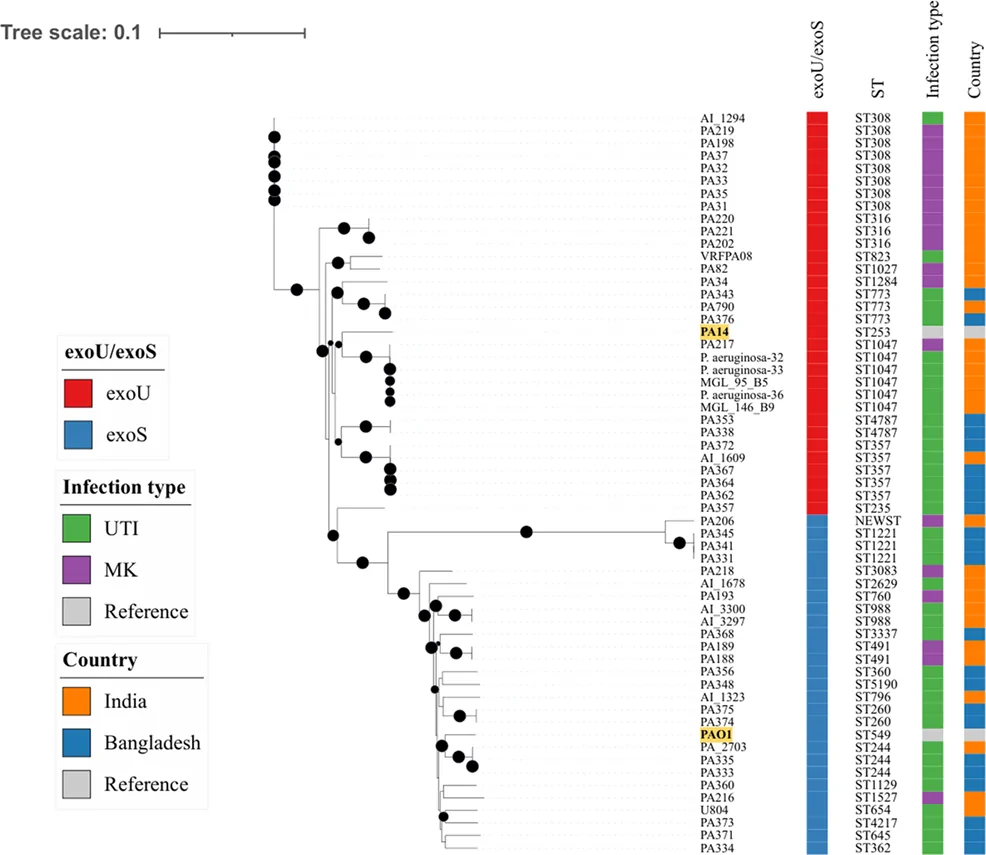
Maximum-likelihood phylogeny of *exoU*- and *exoS*-carrying *P. aeruginosa* isolates from urinary tract infections (UTIs) and microbial keratitis (MK), inferred from recombination-filtered core-genome SNPs and midpoint-rooted. Branch lengths represent nucleotide substitutions per site, and filled black circles at internal nodes indicate ultrafast bootstrap support ≥ 90%. The dataset comprises 57 clinical isolates (23 UTI isolates from Bangladesh, 15 UTI isolates from India, and 19 MK isolates from India) and two reference strains, *P. aeruginosa* PA14 (*exoU*) and PAO1 (*exoS*), which are highlighted in yellow. Annotation tracks, from left to right, denote *exoU*/*exoS* genotype (*exoU*, red; *exoS*, blue), ST, infection type (UTI, green; MK, purple; reference strains, grey), and country of origin (India, orange; Bangladesh, blue; reference strains, grey)

MLST analysis demonstrated substantial sequence-type diversity among the 57 study isolates. The 31 *exoU*-positive isolates represented 10 STs (ST308, ST316, ST823, ST1027, ST1284, ST773, ST1047, ST4787, ST357, and ST235), whereas the 26 *exoS*-positive isolates represented 19 STs (NEWST, ST1221, ST3083, ST2629, ST760, ST988, ST3337, ST491, ST360, ST5190, ST796, ST260, ST244, ST1129, ST1527, ST654, ST4217, ST645, and ST362). No ST was shared between the *exoU*- and *exoS*-positive study isolates. UTI isolates were highly diverse (24 STs among 38 isolates), whereas MK isolates showed a more restricted ST distribution. Core-genome SNP phylogenetic analysis showed broad segregation according to *exoU*/*exoS* status (Fig. 2), with *exoU*-positive and *exoS*-positive isolates largely distributed into separate phylogenetic group. Both infection types (UTI and MK) and geographic origins (Bangladesh and India) were represented within these major genotype-associated groups. The *exoU*-positive reference strain PA14 clustered with the *exoU*-associated group, whereas *exoS*-positive PAO1 clustered with the *exoS*-associated group. The phylogeny also revealed sequence-type-associated clustering, with ST308, ST1047 and ST357 forming distinct groups within the *exoU* clade, while ST244 and ST1221 were concentrated in the *exoS* clade. The two genotype-associated clades comprised isolates from both Bangladesh and India, demonstrating a geographically mixed distribution. 

**Fig. 3:**
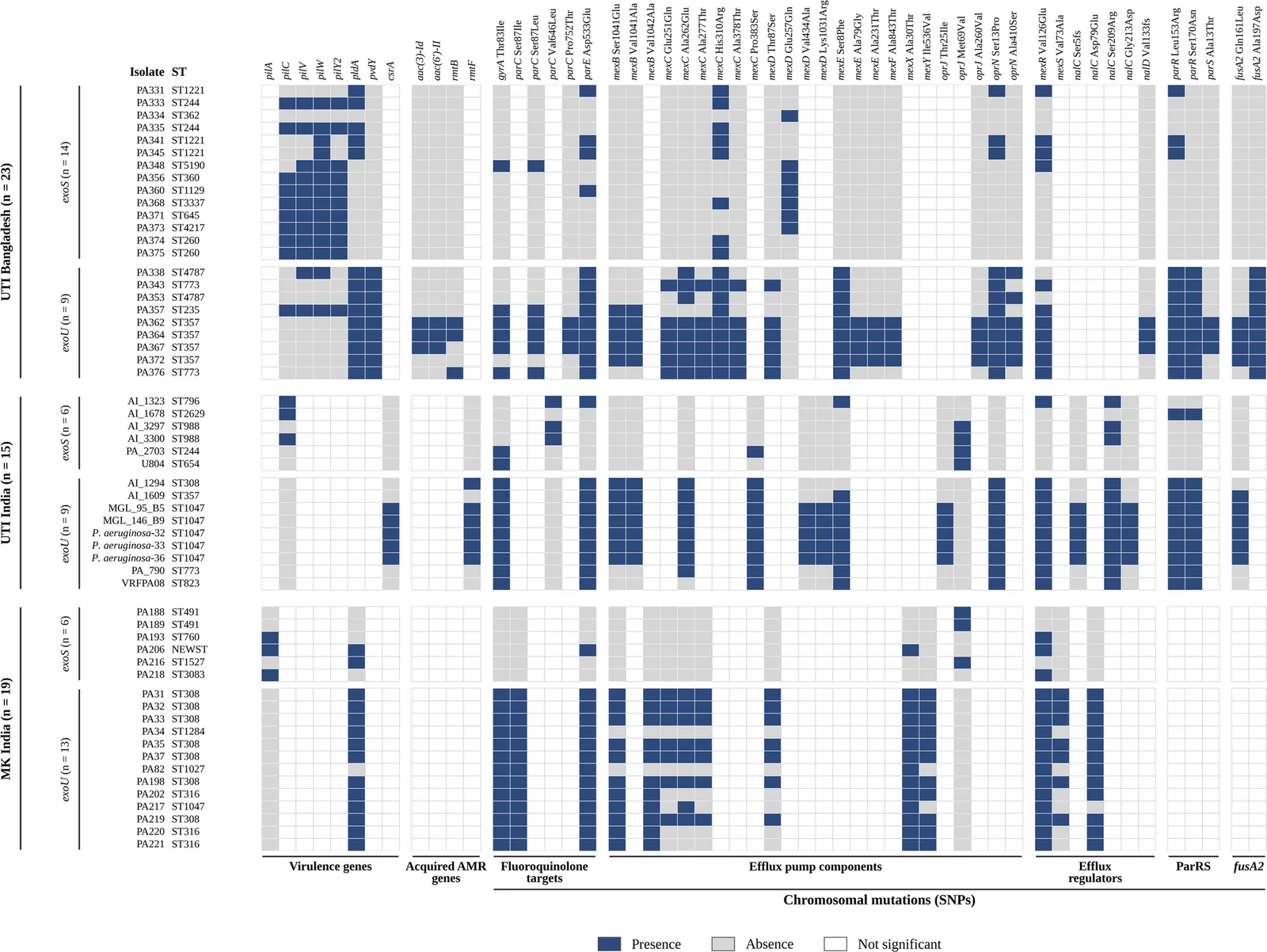
Heatmap showing the isolate-level distribution of resistance-associated chromosomal SNPs, acquired antimicrobial resistance genes, and virulence determinants that differed significantly in prevalence between *exoU*- and *exoS*-positive isolates across the Bangladeshi UTI, Indian UTI, and Indian microbial keratitis (MK) cohorts. Isolates are arranged by cohort, T3SS genotype, and multilocus sequence type (ST). Dark blue indicates feature presence, grey indicates absence, and white indicates features that were not significantly different between *exoU*- and *exoS*-positive isolates in the corresponding cohort. The heatmap illustrates features showing broader *exoU*- or *exoS*-associated distributions across multiple STs, as well as features concentrated within particular STs or clonal clusters, providing ST/clonal context for the observed genotype-associated patterns

Feature-level inspection of all SNPs and acquired resistance genes showing significantly different prevalence between *exoU*- and *exoS*-positive isolates, together with significantly different virulence determinants, in relation to MLST revealed both broad genotype-associated and ST-restricted/cluster-associated patterns (Fig. 3; Table 14).

In the Bangladesh UTI cohort, several *exoU*-associated mutations, MexC Ala262Glu, Ala277Thr, Ala378Thr and Glu251Gln, MexD Thr87Ser, MexE Ser8Phe, OprN Ala410Ser, FusA2 Ala197Asp and ParR Ser170Asn, occurred across multiple *exoU* STs, whereas ParC Pro752Thr, MexE Ala231Thr and Ala79Gly, MexF Ala843Thr, OprJ Ala260Val, NalD Val133fs, FusA2 Gln161Leu, and ParS Ala13Thr were confined to ST357. MexB Ser1041Glu and Val1041Ala were also concentrated within ST357. Conversely, MexD Glu257Gln was distributed across multiple *exoS* STs. The virulence genes *pilC*, *pilV*, *pilW*, and *pilY2* were enriched in *exoS* isolates across several STs, whereas *pldA* and *pvdY* were enriched in *exoU* isolates across multiple STs. Among the significantly different acquired resistance genes, *aac(6′)-II* and *aac(3)-Id* were restricted to ST357 *exoU*-positive isolates, indicating an ST-associated contribution to their apparent *exoU* association. *rmtB* was detected in two *exoU* STs (ST357 and ST773), although two of the three positive isolates belonged to ST357, suggesting partial ST concentration rather than restriction to a single ST.

In the Indian UTI cohort, MexB Ser1041Glu and Val1041Ala, MexC Ala262Glu and OprN Ser13Pro were present across several *exoU* STs, supporting a broader *exoU*-associated distribution. The *exoS*-associated OprJ Met69Val and ParC Val646Leu were distributed across multiple *exoS* STs. In contrast, MexD Lys1031Arg and Val434Ala, NalC Gly213Asp and Ser5fs, and OprJ Thr25Ile were restricted to the ST1047 *exoU* cluster. FusA2 Gln161Leu was also concentrated within ST1047, although one positive isolate belonged to another ST. The virulence gene *csrA* was also concentrated in ST1047 *exoU* isolates, whereas *pilC* was detected in three different *exoS* STs. The acquired aminoglycoside resistance gene *rmtF* was detected across two *exoU* STs (ST1047 and ST308); however, five of the six *rmtF*-positive isolates belonged to ST1047, indicating an ST1047-associated contribution to its apparent *exoU* enrichment.

Among Indian MK isolates, GyrA Thr83Ile, ParC Ser87Ile, MexB Ser1041Glu and Val1042Ala, NalC Asp79Glu, and MexY Ile536Val were distributed across multiple *exoU* STs. The *exoS*-associated OprJ Met69Val was distributed across multiple *exoS* STs. By contrast, MexC Ala277Thr, MexC Glu251Gln, MexD Thr87Ser, and MexS Val73Ala were restricted to the ST308 cluster, indicating a ST-restricted/cluster-associated pattern. MexC Ala262Glu was also concentrated within ST308, with seven of eight positive isolates belonging to ST308. The *exoS*-associated virulence gene *pilA* occurred in three distinct *exoS* STs, while *pldA* was enriched among *exoU* isolates distributed across several STs. No acquired resistance gene showed significantly different prevalence between *exoU*- and *exoS*-positive isolates in the Indian MK cohort.

Several additional mutations were significantly enriched in *exoU*-positive isolates but were not genotype-specific, as they were also detected at lower frequencies in *exoS*-positive isolates. In the Bangladesh UTI cohort, these included GyrA Thr83Ile, ParC Ser87Leu, ParE Asp533Glu, MexC His310Arg, MexR Val126Glu, OprN Ser13Pro, and ParR Leu153Arg. In the Indian UTI cohort, GyrA Thr83Ile, ParE Asp533Glu, MexC Pro383Ser, MexE Ser8Phe, MexR Val126Glu, NalC Ser209Arg, and ParR Leu153Arg and Ser170Asn showed the same pattern. In the Indian MK cohort, this pattern was observed for ParE Asp533Glu, MexR Val126Glu, and MexX Ala30Thr. These mutations were distributed across multiple STs, supporting broader genotype-associated rather than ST-restricted patterns.

To assess whether the observed variation in MMR-genes (*mutL*, *mutS* and *uvrD*) could be influenced by clonal background, isolate-level MMR SNP burden was examined in relation to ST and the core-genome phylogeny (Supplementary Table 15). In the Bangladeshi UTI cohort, elevated MMR SNP counts (5–7 SNPs) were concentrated in two STs: ST4787 (PA338 and PA353; 7 SNPs each) and ST357 (PA362, PA364, PA367, and PA372; 5 SNPs each). In the Indian UTI cohort, five ST1047 isolates each carried 7 MMR-gene SNPs, indicating a clear within-ST pattern. However, elevated MMR SNP counts were also observed in genetically distinct Indian STs, including ST308, ST357, ST773, and ST823. Thus, elevated MMR variation showed evidence of clonal/ST structure but was not restricted to a single clone.

Overall, the presence of multiple STs within both *exoU*- and *exoS*-positive groups indicates substantial genetic diversity within each genotype and suggests that the broad *exoU*/*exoS* phylogenetic segregation was not attributable solely to expansion of a single clone. However, the restriction or concentration of several resistance-associated variants, acquired resistance genes, virulence determinants, and elevated MMR SNP burdens within specific STs, including ST357, ST1047, and ST308, indicates that ST/clonal background may have contributed to a subset of the observed associations.

**Table 14:** Phylogenetic context of resistance-associated SNPs, acquired resistance genes, and virulence-factor genes showing significantly different prevalence between *exoU*- and *exoS*-positive isolates across the three study cohorts

Cohort	Features distributed across multiple STs (broader *exoU*/*exoS*-associated pattern)	Features concentrated within a particular ST/cluster (possible ST/clonal contribution)
Bangladeshi UTI	*exoU*-associated SNPs: MexC Ala262Glu, Ala277Thr, Ala378Thr and Glu251Gln, MexD Thr87Ser, MexE Ser8Phe; OprN Ala410Ser, FusA2 Ala197Asp, ParR Ser170Asn. *exoS*-associated SNP: MexD Glu257Gln. Virulence factors: *pilC*, *pilV*, *pilW* and *pilY2* were enriched in *exoS*-positive isolates across multiple STs, whereas *pldA* and *pvdY* were enriched in *exoU*-positive isolates across multiple STs.	ST357-associated: ParC Pro752Thr, MexE Ala231Thr and Ala79Gly, MexF Ala843Thr, NalD Val133fs, OprJ Ala260Val, FusA2 Gln161Leu, ParS Ala13Thr. ST357 concentration: MexB Ser1041Glu and Val1041Ala. Acquired resistance genes: *aac(6′)-II* and *aac(3)-Id* were restricted to ST357; *rmtB* was concentrated in ST357, with one positive isolate belonging to ST773.
Indian UTI	*exoU*-associated SNPs: MexB Ser1041Glu and Val1041Ala, MexC Ala262Glu, OprN Ser13Pro. *exoS*-associated SNPs: OprJ Met69Val, ParC Val646Leu. Virulence factor: *pilC* was associated with *exoS*-positive isolates across multiple STs.	ST1047-associated: MexD Lys1031Arg and Val434Ala; NalC Gly213Asp and Ser5fs, OprJ Thr25Ile. ST1047 concentration: FusA2 Gln161Leu, although one positive isolate belonged to another ST. Virulence factor: *csrA* was concentrated in ST1047 *exoU*-positive isolates. Acquired resistance gene: *rmtF* showed ST1047 concentration, with five of six positive isolates belonging to ST1047 and one to ST308.
Indian MK	*exoU*-associated SNPs: GyrA Thr83Ile; ParC Ser87Ile; MexB Ser1041Glu and Val1042Ala, MexY Ile536Val, NalC Asp79Glu. *exoS*-associated SNP: OprJ Met69Val. Virulence factors: *pilA* was associated with *exoS*-positive isolates across multiple STs, whereas *pldA* was enriched in *exoU*-positive isolates across multiple STs. Acquired resistance genes: none showed significantly different prevalence between *exoU*- and *exoS*-positive isolates.	ST308-associated: MexC Ala277Thr and Glu251Gln, MexD Thr87Ser, MexS Val73Ala. ST308 concentration: MexC Ala262Glu, with seven of eight positive isolates belonging to ST308.

Features distributed across multiple STs were interpreted as broader *exoU/exoS*-associated patterns, whereas features concentrated within a particular ST or phylogenetic cluster were considered potentially influenced by ST/clonal background.

## Discussion

The present study aimed to differentiate phenotypic and genomic determinants of antibiotic resistance and key virulence factors of *exoU* and *exoS* in *P. aeruginosa* UTI isolates from Bangladesh, followed by genotypic comparison with Indian UTI isolates. Overall, the prevalence of *exoS* isolates was 60.9%, which was higher than *exoU* (39.1%) in Bangladesh. In contrast, *exoU* was more prevalent than *exoS* among the Indian UTI isolates (60.0% vs. 40.0%), with no significant difference between the two UTI cohorts. Previous studies on UTI isolates showed a higher prevalence of *exoS* strains at 67.3–100% compared to *exoU* at 4-10.2% (Elbargisy 2022; de Sousa et al. 2023). However, the opposite trend was also observed in other studies with *exoU* at 66.2–94.8% compared to 29.4–34.5% *exoS* (Park and Koo 2022; Cho and Park 2025).

In Bangladeshi UTI isolates, the *exoU* strains showed a higher antibiotic resistance compared to *exoS* isolates against all four tested antibiotics (ciprofloxacin, levofloxacin, gentamicin, and tobramycin). A previous study with UTI isolates reported that *exoU* was strongly associated with the multidrug-resistant (MDR) phenotype (Elbargisy 2022). Moreover, studies on clinical samples collected from ocular infections, cystic fibrosis, bacteremia and respiratory tract infections also found a higher association between *exoU* strains and resistance phenotype, against similar antibiotics (Hassuna 2016; Park et al. 2017; Subedi et al. 2018a).

Analysis of SNPs in target-site, efflux pump, and their regulatory genes, along with possession of acquired resistance genes of Bangladeshi and Indian UTI isolates revealed several SNPs and presence of resistance genes, some of which have previously been reported to be associated with antibiotic resistance. In particular, GyrA Thr83Ile and ParC Ser87Leu mutations in Bangladeshi samples showed strongest association (*p*_*adj*_*=* 0.02) with the fluoroquinolone antibiotic resistance. These SNPs affect the QRDR region by altering the structures of DNA gyrase and topoisomerase IV, reducing fluoroquinolone binding, and thereby preventing antibiotics from effectively acting on the DNA (Hooper and Jacoby 2015; Arabameri et al. 2021). Studies showed that the presence of these mutations in GyrA and ParC in isolates were highly resistant to fluoroquinolone antibiotics aligning with current study (Arabameri et al. 2021; El-Badawy et al. 2022). Moreover, a study performed in Bangladesh with a variety of clinical isolates including UTI, showed the presence of GyrA Thr83Ile in all isolates while ParC Ser87Leu mutation in 20% isolates indicating the high prevalence of these mutations in Bangladeshi *P. aeruginosa* isolates (Arefin et al. 2025). Current results also indicated that, these mutations were significantly associated (*p*_*adj*_*=* 0.02) with the *exoU* isolates which is similar to the previous studies (Subedi et al. 2018a; Akter et al. 2025). Another mutation, Asp533Glu in ParE was also significantly correlated (*p*_*adj*_*<* 0.01) with both *exoU* strains and fluoroquinolone resistance (*p*_*adj*_*=* 0.04) which also aligned with the previous findings (Varga et al. 2015; Subedi et al. 2018a; Akter et al. 2025; Roy et al. 2026). The mutation His148Asn in GyrB was only identified in one fluoroquinolone-resistant PA360. However, previous studies have suggested that the association of GyrB His148Asn with fluoroquinolone resistance is ambiguous, and its independent contribution to the resistant phenotype remains uncertain (Oh et al. 2003). Although PA360 lacked the canonical GyrA Thr83Ile and ParC Ser87Leu substitutions, it carried ParE Asp533Glu together with variants in efflux-associated regulatory genes, including NalC Gly71Glu and Ser209Arg and MexT Gln80fs, as well as several variants in the MexCD-OprJ efflux system. These alterations may contribute to the atypical fluoroquinolone-resistant phenotype of PA360; however, their individual functional contributions remain unconfirmed and require functional and expression-based validation. When comparing Bangladeshi and Indian isolates, both regions shared an *exoU*-associated resistome consisting of GyrA Thr83Ile and ParE Asp533Glu. This is supportive of previous literature identifying *exoU* to be associated with higher levels of quinolone and multidrug resistance in nosocomial infections (Subedi et al. 2018c; Horna et al. 2019; Akter et al. 2025).

In addition to the QRDR SNPs, mutations were observed in the Resistance-Nodulation Division (RND) efflux systems. Mutations affecting these efflux pumps components and their regulators can alter the efflux pumps overexpression, leading to increased antibiotic resistance (Hocquet et al. 2007). In the current findings, the most significant mutation was Val126Glu in MexR, the regulator of MexAB-OprM. This mutation showed a significant positive correlation (*p*_*adj*_*=* 0.04) with fluoroquinolone resistance in Bangladeshi UTI isolates. This finding corroborated previous results showing similar association (Gorgani et al. 2009; Akter et al. 2026a). Moreover, The MexR Val126Glu mutation was significantly more frequent in *exoU* isolates from both the Bangladeshi and Indian cohorts. The exclusive presence of NalC Gly213Asp and Ser5fs mutations among the Indian isolates might be associated with repressor of the MexAB-OprM pump as they are solely present in *exoU* isolates, however further studies are warranted to confirm this. All differentially prevalent SNPs in *mexE*, *mexF* and *oprN* between *exoU* and *exoS* Bangladeshi isolates were significantly associated with (*p*_*adj*_ < 0.05) *exoU* strains but only MexE Ser8Phe had association with fluoroquinolone resistance (φ = 0.55; *p*_*adj*_ = 0.04). In particular, the Ser8Phe mutation in MexE showed the strongest association with resistance, which is in contrast with the previous study, where no relation was found (Akter et al. 2025). No mutation was detected in the *mexX*,* mexY*, and *mexS* genes in any isolates from both countries.

In the case of aminoglycoside resistance, both acquired AMEs and SNPs in regulatory/efflux pumps were prevalent across Bangladeshi isolates with elevated MICs to gentamicin and tobramycin, Among the AMEs, the genes *aac(6′)-II*, *aac(3)-Id*, and *rmtB* were detected (*p*_*adj*_*=* 0.05) only in *exoU* Bangladeshi isolates (33%). A previous study in Bangladesh also showed the presence of *aac(6′)-II* in gene was associated with resistance in clinical and environmental isolates (Zahra et al. 2022). This gene specifically acetylates gentamicin, rendering them ineffective (Rather et al. 1992). Although *rmtB* was not significantly associated with aminoglycoside resistance, all three *rmtB*-positive isolates exhibited gentamicin and tobramycin MICs of ≥ 5120 µg/mL. This apparent discrepancy may reflect the fact that resistance was assessed as a categorical phenotype, which does not capture differences in the magnitude of MICs. In addition, the small number of *rmtB*-positive isolates limited the statistical power to detect an association. Therefore, the absence of statistical significance does not necessarily indicate limited biological relevance, particularly given the consistently extreme MICs observed among *rmtB*-positive isolates. However, because the *rmtB*-positive isolates also carried additional acquired resistance genes and SNPs in *fusA2*, *parR*, and *parS*, their extreme resistance phenotype cannot be attributed solely to *rmtB*. In contrast, PA357, PA367, and PA338 also exhibited tobramycin MICs of ≥ 5120 µg/mL without a detected 16S rRNA methylase gene. These isolates carried other acquired aminoglycoside-resistance genes together with SNPs in *fusA2*, *parR*, and *parS*. Collectively, these findings suggest that multiple acquired resistance genes and resistance-associated SNPs may contribute to extreme aminoglycoside resistance, although their individual contributions cannot be resolved from the present genomic data. PA348 was the only *exoS* isolate exclusively possessed *rmtF*, the 16S rRNA methyltransferase gene and the adenyltransferase *ant(4′)-IIb* along with an *exoU* isolate PA367 and both strains showed high resistance to both gentamicin and tobramycin. These findings suggest that these AME genes may play a significant role in aminoglycoside resistance of these isolates. As compared to the Bangladeshi isolates, the Indian isolates uniquely carried *aph(3′)-VIa*, *ant(2′′)-Ia*, and *rmtD2*, whereas *aac(3)-Ic*, *ant(4′)-IIb*, and *aadA6* were unique to the Bangladeshi isolates. All of these AME genes have previously been reported to be associated with aminoglycoside resistance in *P. aeruginosa*. (Riccio et al. 2003; Sabtcheva et al. 2003; Poole 2005; Vaziri et al. 2011; Subedi et al. 2018d; Thirumalmuthu et al. 2019; Akter et al. 2025).

In addition to the AMEs, a few SNPs in genes *fusA2* and *parR* in Bangladeshi UTI isolates showed association with aminoglycosides resistance. In ParR, the Ser170Asn mutation was significantly associated with tobramycin resistance, consistent with previous findings (Akter et al. 2025). This SNP was also detected in Indian UTI isolates. FusA2 Ala197Asp was associated with tobramycin resistance in Bangladeshi *exoU* UTI isolates. These findings suggest that variation in ParR and FusA2 may contribute to tobramycin resistance in *exoU P. aeruginosa* UTI isolates. Notably, MLST analysis showed that FusA2 Ala197Asp occurred across multiple *exoU* STs in the Bangladeshi UTI cohort, suggesting that its association was not solely attributable to expansion of a single clone. Nevertheless, its absence as a differentially prevalent variant in the Indian UTI cohort suggests that it is better regarded as a cohort-specific *exoU*-associated variant than as a universal UTI-niche marker.

Study of the SNPs in the DNA mismatch repair (MMR) genes (*mutL*, *mutS* and *uvrD*) showed key findings where the burden of SNPs was higher in *exoU* strains, which may indicate increased mutational propensity. The impairment of the MMR system has been linked to generating hypermutable phenotypes and this hypermutation is a key aspect in the development of antibiotic resistance in *P. aeruginosa* (Macia et al. 2005; Dulanto Chiang et al. 2022). Moreover, the hypermutable phenotype has been linked to chronic infections and multidrug resistance (Oliver et al. 2000; Macia et al. 2005). A study on de novo *mutS*-associated mutational signatures observed similar accumulation of transitions and frameshift mutations (Hall et al. 2025). In the present study, SNP counts in *mutL* in Bangladeshi UTI isolates and in *mutL* and *uvrD* in Indian UTI isolates showed stronger positive correlations with AMR-associated SNP burden than *mutS*. The cohort-specific association of *uvrD* may partly reflect differences in ST composition and the distribution of *uvrD* SNPs between the two populations, as higher *uvrD* SNP burdens were observed across several Indian STs, whereas their distribution was more heterogeneous in the Bangladeshi cohort (Supplementary Table 15). These findings contrast with previous studies reporting a stronger association between *mutS* defects and antimicrobial resistance (Chopra et al. 2003). MLST and core-genome phylogenetic analyses suggest that part of the observed MMR variation may reflect ST/clonal background. As mutation rates and MMR function were not directly assessed, these findings indicate an association between MMR variation and AMR-associated mutational burden, rather than direct evidence of hypermutation. Further mutation-rate and functional studies are needed to clarify the roles of *mutL* and *uvrD*.

Current study also compared antibiotic resistance and virulence factors of Indian keratitis and UTI isolates. Across keratitis and UTI infection sites, both GyrA Thr83Ile and ParE Asp533Glu were consistently present in *exoU* isolates. The divergence of mutations across infection sites is apparent when observing efflux mutations. Mutations in *mexS* were entirely absent in UTI isolates whilst MexS Val73Ala was differentially prevalent in 53.8% of *exoU* isolates of keratitis (Akter et al. 2025). As *mexS* is a MexEF-OprN regulator, mutations here may result in efflux overexpression and increased resistance to fluoroquinolones in keratitis isolates (Akter et al. 2025).

Aminoglycoside-resistance associated mutations also differed across infection sites, with UTI isolates showing higher prevalence of FusA2 Gln161Leu in *exoU* isolates, whilst no mutation on FusA2 was present in keratitis cases. The distribution of acquired resistance genes across infection sites was also distinct, with the *rmtF* gene, encoding a 16S rRNA methylase, being detected predominantly in Indian UTI isolates, whereas the *crpP* gene was present only in Indian keratitis isolates. The presence of *rmtF* in UTI isolates is concerning as it confers high levels of resistance to aminoglycosides, narrowing treatment options for complex UTIs infection (Rahman et al. 2015). Although *rmtF* was enriched in *exoU*-positive Indian UTI isolates, its detection in one Bangladeshi *exoS* isolate and clustering within ST1047 suggest that this association may reflect cohort and clonal background rather than an *exoU*-specific epidemiological marker.

The genotype-stratified virulence gene analysis in this study revealed both conserved and infection-context-specific patterns between *exoU* and *exoS* isolates of *P. aeruginosa* across UTIs and MK. The most consistent finding in both infection types was the enrichment of *pldA* in *exoU* isolates. This gene encodes a phospholipase D enzyme secreted via the hemolysin co-regulated protein secretion island II type VI secretion system (H2-T6SS) (Wettstadt et al. 2019). PldA is a trans-kingdom effector, which kills competitor bacteria by cleaving phosphatidylethanolamine in bacterial cell membranes and facilitates internalization into non-phagocytic host cells via activation of the PI3K/Akt signaling pathway (Russell et al. 2013; Jiang et al. 2014). The *pldA* gene is not universally present across *P. aeruginosa* strains, and its horizontal acquisition is supported by the highly conserved genomic regions flanking an insertion site (Wilderman et al. 2001). Its co-enrichment with *exoU*, observed here in both Bangladeshi UTI and Indian MK isolates (Akter et al. 2026d), mirrors earlier findings linking *pldA* to hypervirulent and multidrug-resistant *exoU* strains in septicemia, pulmonary infections, and UTIs (Boulant et al. 2018). All of these findings suggest that the co-occurrence of *exoU* and *pldA* may act synergistically, thereby enhancing tissue destruction and bacterial fitness in both ocular and urinary infection sites, although this potential interaction requires further confirmation.

Type IV pili (T4P) are essential for *P. aeruginosa* pathogenesis by driving twitching motility and they mediate binding to asialo-GM1 receptors on host epithelial cells (Akter et al. 2026a). The major pilin subunit PilA is specifically required for the injection of ExoS into host cells via pilus retraction, directly coupling T4P function to T3SS effector delivery in *exoS* strains (Hayashi et al. 2015; Shikata et al. 2016). This mechanistic link may explain why *pilA* and other pilus biosynthesis genes (*pilC*, *pilV*, *pilW*, *pilY2*) were consistently enriched in *exoS* isolates in the MK and UTI dataset respectively. *exoS* strains appear to rely more heavily on pilus-mediated attachment and internalization, possibly compensating for the absence of the rapid, phospholipase-driven cytotoxicity conferred by *exoU*. In the urinary tract, T4P-mediated adhesion to uroepithelial surfaces may further amplify the significance of this genotype-pili association in the UTIs (Nieto et al. 2019). In the current study, *pilC* was detected in both Bangladeshi and Indian isolates. The reduced carriage of T4P biosynthesis genes in *exoU* isolates may reflect a distinct virulence strategy rather than directly indicating immune evasion or a fitness cost. As pili function and host immune interactions were not experimentally assessed in *exoU* isolates, the biological consequence of this pattern remains uncertain.

Pyoverdine is the primary siderophore and it is a well-established virulence determinant in *P. aeruginosa* (Peek et al. 2012; Ghssein and Ezzeddine 2022). In iron-limited host environments, it acquires ferric iron from host proteins such as transferrin and lactoferrin and additionally acts as a signaling molecule that upregulates other virulence factors, including exotoxin A and the endoprotease PrpL (Bonneau et al. 2020). Strains lacking pyoverdine show reduced virulence in multiple infection models (Bonneau et al. 2020). The differential carriage of pyoverdine biosynthesis gene, *pvdY*, was enriched in *exoU* strains in Bangladesh UTI cohort. The iron-limited environment of the urinary tract makes pyoverdine-mediated iron acquisition particularly important in UTI pathogenesis (Mittal et al. 2009). This UTI-specific observation warrants further investigation. In contrast, pyoverdine gene was not identified as discriminatory between genotypes in the MK dataset, which may reflect differences in iron availability on the ocular surface.

The carbon storage regulator A (*csrA*) encodes a post-transcriptional RNA-binding regulator that governs carbon metabolism, motility, biofilm formation, and quorum sensing, and acts as a global activator of acute-phase virulence traits, including T3SS expression and T4P-mediated motility (Gutierrez et al. 2005; Rojano-Nisimura et al. 2024). Notably, RsmA, the *P. aeruginosa* homolog of CsrA, positively regulates T3SS effector delivery and acute infection phenotypes while repressing biofilm formation and T6SS, thereby controlling the transition between acute and chronic infection models (Marden et al. 2013; Allsopp et al. 2017). In the Indian UTI cohort, *csrA* was detected only in *exoU* isolates; however, MLST analysis showed that it was concentrated within ST1047, suggesting that its apparent *exoU* association may partly reflect clonal background. Its absence as a discriminatory virulence determinant in the MK cohort may indicate an infection-site-specific pattern, although this requires further confirmation.

MLST and core-genome phylogenetic analyses showed that several resistance and virulence features were distributed across multiple STs, supporting broader *exoU*- or *exoS*-associated patterns, whereas others were concentrated within specific STs, particularly ST357, ST1047, and ST308. Elevated MMR SNP burdens also showed partial ST clustering but were not restricted to a single ST. Together, these findings indicate that both broader genotype-associated patterns and ST/clonal background may have contributed to the observed differences. Future studies using larger, geographically diverse isolate collections and population-structure-adjusted genomic analyses will be needed to determine whether the observed *exoU/exoS*-associated differences remain significant after controlling for ST and clonal population structure.

From a clinical and epidemiological perspective, the association of *exoU*-positive *P. aeruginosa* isolates with increased resistance to commonly used antipseudomonal antibiotics may have implications for the management and surveillance of UTIs and MK. These findings highlight the importance of antimicrobial susceptibility testing and suggest that *exoU* status, together with resistance-associated genetic markers, may provide additional epidemiological information for identifying and monitoring isolates with more resistant phenotypes. The observed differences in resistance and virulence-associated characteristics between *exoU*- and *exoS*-positive isolates may also contribute to molecular surveillance and the recognition of potentially high-risk circulating strains. MLST and core-genome phylogenetic analyses further underscore the importance of strain typing when assessing possible clonal dissemination or outbreak-related transmission.

This study has several limitations. First, the relatively small sample sizes, particularly within the genotype-stratified groups, may limit the statistical power to detect less frequent differences and the generalizability of the findings. Second, the study included isolates from only Bangladesh and India; therefore, the observed geographic patterns may not represent *P. aeruginosa* populations from other regions. Third, the virulence analysis was based on the presence of virulence-associated genes, and their phenotypic expression or functional activity was not experimentally assessed. Similarly, although several genetic variants were associated with antimicrobial resistance phenotypes, the functional effects of individual variants were not experimentally validated, and these associations should not be interpreted as evidence of causality. In addition, despite the use of the same quality assessment, annotation, and downstream analysis pipeline, potential batch effects cannot be completely excluded because the Bangladeshi isolates were newly sequenced and assembled, whereas the Indian genomes were obtained as publicly available assembled sequences. Differences in library preparation, sequencing conditions, or original assembly procedures may therefore have contributed to some of the observed geographic variation and should be considered when interpreting comparisons between the two cohorts. Finally, the cross-sectional design and absence of detailed clinical outcome data prevented assessment of whether the identified resistance and virulence-associated characteristics were associated with disease severity, treatment response, or patient outcomes. Larger studies incorporating functional validation and linked clinical data are warranted to confirm and extend these findings.

## Conclusions

*exoU* UTI isolates from Bangladesh showed higher resistance to fluoroquinolones and aminoglycosides than *exoS* isolates. More broadly, comparisons across Bangladeshi and Indian UTI isolates, together with Indian UTI and MK isolates, showed that *exoU* isolates consistently carried a greater burden of resistance-associated mutations across regions and infection sites. Core mutations commonly enriched in *exoU* isolates included GyrA Thr83Ile, ParE Asp533Glu, MexR Val126Glu, MexB Ser1041Glu, MexC Ala262Glu. Acquired resistance genes also showed regional and niche-specific patterns, with *aac(6′)-II* and *aac(3)-Id* more common in Bangladeshi UTI isolates, *rmtF* associated with Indian UTI isolates, and *crpP* restricted to Indian MK isolates. Virulence profiling further demonstrated both shared and site-specific differences between *exoU* and *exoS* isolates, with *exoU* isolates more often associated with enzyme- and iron uptake-related genes, whereas *exoS* isolates were more frequently associated with type IV pili-associated adherence genes. MLST and core-genome phylogenetic analyses indicated that many of the observed differences were broadly associated with *exoU* or *exoS* across multiple STs, although ST/clonal background may also have influenced a subset of these associations. Together, these findings indicate that *exoU* and *exoS* isolates retain distinct core resistance and virulence signatures, while also showing regional and infection-specific, and clonal variation.

## Electronic Supplementary Material

Below is the link to the electronic supplementary material.

Supplementary Material 1 (DOCX 18 KB)

Supplementary Material 2 (DOCX 18 KB)

Supplementary Material 3 (DOCX 18 KB)

Supplementary Material 4 (DOCX 30 KB)

Supplementary Material 5 (DOCX 21 KB)

Supplementary Material 6 (DOCX 30 KB)

Supplementary Material 7 (DOCX 18 KB)

Supplementary Material 8 (DOCX 22 KB)

Supplementary Material 9 (DOCX 28 KB)

Supplementary Material 10 (DOCX 21 KB)

Supplementary Material 11 (DOCX 28 KB)

Supplementary Material 12 (DOCX 19 KB)

Supplementary Material 13 (XLSX 73 KB)

Supplementary Material 14 (XLSX 61 KB)

Supplementary Material 15 (DOCX 33 KB)

## Data Availability

Data will be made available on request.
