# Supplementary material for: Comparative study of antibiotic resistance and virulence profiles of exoU and exoS Pseudomonas aeruginosa isolates from urinary tract infections and microbial keratitis in Bangladesh and India
Source: World J Microbiol Biotechnol. 2026 Oct 12;42(10):602. doi: 10.1007/s11274-026-05320-2 (PMC13658543; doi:10.1007/s11274-026-05320-2)
Supplement: Supplementary file 1 [file 11274_2026_5320_MOESM10_ESM.docx]

Supplementary Table 10: The mutations in fluoroquinolone resistance genes that were not significantly different between 9 *exoU* and 6 *exoS* UTI isolates from India

| Gene | Mutation |
| --- | --- |
| *gyrA* | Tyr100Cys, Val671Ile, Gly860Ser, Asp893Glu, Ala900Gly, Ser903Ala |
| *gyrB* | Glu468Asp |
| *parC* | Ser87Leu, Ala88Pro, Ala639Gly, Asp645Gly, Pro752Thr |
| *parE* | Asp142Asn, Val125fs |
| *mexA* | Asp373Asn |
| *mexB* | Gly957Asp |
| *oprM* | Ala309fs |
| *mexC* | Arg76Gln, Thr175Ala, Glu251Gln, Ala277Thr, His310Arg, Ser330Ala, Glu347Lys, Ala378Thr, Ala384Val |
| *mexD* | Thr87Ser, Glu257Gln, Thr286Met, Ser845Ala |
| *oprJ* | Asp68Gly, Arg168Trp, Arg210His, Ala260Val |
| *mexE* | Ala79Gly, Ala231Thr, Asp353Glu, Asp370Glu |
| *mexF* | Asp230Ala, Ala550Val, Asp666Glu, Ala843Thr, Glu1008fs |
| *oprN* | Ala38Thr, Asp31Val, Ala410Ser, Val402Gly, Ser403Pro |
| *nalC* | Gly71Glu, Asp79Glu, Ala186Thr |
| *nalD* | Gly81fs, Val133fs, Val151fs, Leu153Gln |
| *mexT* | Arg40His, Pro60Ser, Gln80fs, Ala143Thr, Phe172Ile |
| *nfxB* | Ser5Phe |

Abbreviations: fs, frameshift mutation
