# Supplementary material for: Comparative study of antibiotic resistance and virulence profiles of exoU and exoS Pseudomonas aeruginosa isolates from urinary tract infections and microbial keratitis in Bangladesh and India
Source: World J Microbiol Biotechnol. 2026 Oct 12;42(10):602. doi: 10.1007/s11274-026-05320-2 (PMC13658543; doi:10.1007/s11274-026-05320-2)
Supplement: Supplementary file 2 [file 11274_2026_5320_MOESM11_ESM.docx]

Supplementary Table 11. Possession of acquired resistance genes and functional mutations in genes associated with aminoglycoside resistance of 9 *exoU* and 6 *exoS* UTI isolates from India

| Strain ID | TTSS | *aph(3')-IIb* | *aph(3')-VIa* | *aph(3'')-Ib* | *aph(6)- Id* | *aac(6')-II* | *aac(3)-Id* | *aadA11* | *ant(2'')- Ia* | *rmtD2* | *rmtB* | *rmtF* | *armZ* | *fusA2* | *parR* | *parS* |
| --- | --- | --- | --- | --- | --- | --- | --- | --- | --- | --- | --- | --- | --- | --- | --- | --- |
| AI_1323 | *exoS* |  |  |  |  |  |  |  |  |  |  |  |  | Ser176Ala, Gly695Ala |  | His398Arg |
| AI_1678 | *exoS* |  |  |  |  |  |  |  |  |  |  |  |  | Ser176Ala, Lys306Asn, Gly695Ala | Ser170Asn,  Leu153Arg | His398Arg,  Val313Ile,  Arg243His |
| AI_3297 | *exoS* |  |  |  |  |  |  |  |  |  |  |  |  | Ser176Ala,  Ala197Asp,  Gln601His,  Gly695Ala |  | His398Arg |
| AI_3300 | *exoS* |  |  |  |  |  |  |  |  |  |  |  |  | Ser176Ala  Ala197Asp,  Gln601His,  Gly695Ala |  | His398Arg |
| PA_2703 | *exoS* |  |  |  |  |  |  |  |  |  |  |  |  | Gly695Ala |  | His398Arg |
| U804 | *exoS* |  |  |  |  |  |  |  |  |  |  |  |  |  |  | His398Arg |
| AI_1294 | *exoU* |  |  |  |  |  |  |  |  |  |  |  |  | Ser176Ala, Ala197Asp, Gly695Ala | Ser170Asn,  Leu153Arg | His398Arg |
| AI_1609 | *exoU* |  |  |  |  |  |  |  |  |  |  |  |  | Gln161Leu, Ser176Ala, Ala197Asp, Gly695Ala | Ser170Asn,  Leu153Arg | His398Arg,  Ala13Thr |
| MGL_95_B5 | *exoU* |  |  |  |  |  |  |  |  |  |  |  |  | Gln161Leu,  Ser176Ala,  Ala197Asp,  Gly695Ala | Ser170Asn,  Leu153Arg | His398Arg |
| MGL_146_B9 | *exoU* |  |  |  |  |  |  |  |  |  |  |  |  | Gln161Leu,  Ser176Ala,  Ala197Asp,  Gly695Ala | Ser170Asn,  Leu153Arg | His398Arg |
| P.aeruginosa-32 | *exoU* |  |  |  |  |  |  |  |  |  |  |  |  | Gln161Leu,  Ser176Ala,  Ala197Asp,  Gly695Ala | Ser170Asn,  Leu153Arg | His398Arg |
| P.aeruginosa-33 | *exoU* |  |  |  |  |  |  |  |  |  |  |  |  | Gln161Leu,  Ser176Ala,  Ala197Asp,  Gly695Ala | Ser170Asn,  Leu153Arg | His398Arg |
| P.aeruginosa-36 | *exoU* |  |  |  |  |  |  |  |  |  |  |  |  | Gln161Leu,  Ser176Ala,  Ala197Asp,  Gly695Ala | Ser170Asn,  Leu153Arg | His398Arg |
| PA790 | *exoU* |  |  |  |  |  |  |  |  |  |  |  |  | Ser176Ala,  Ala197Asp,  Gly695Ala | Ser170Asn,  Leu153Arg | His398Arg |
| VRFPA08 | *exoU* |  |  |  |  |  |  |  |  |  |  |  |  | Ser176Ala,  Lys573Thr,  Gly695Ala | Ser170Asn,  Leu153Arg | His398Arg,  Ala395Thr |

Red indicates functional mutations; Dark Box indicates presence of acquired resistance gene.
