# Supplementary material for: Comparative study of antibiotic resistance and virulence profiles of exoU and exoS Pseudomonas aeruginosa isolates from urinary tract infections and microbial keratitis in Bangladesh and India
Source: World J Microbiol Biotechnol. 2026 Oct 12;42(10):602. doi: 10.1007/s11274-026-05320-2 (PMC13658543; doi:10.1007/s11274-026-05320-2)
Supplement: Supplementary file 3 [file 11274_2026_5320_MOESM12_ESM.docx]

**Supplementary Table 12**: Mutations in the DNA mismatch repair (MMR) system genes of 15 UTI isolates (9 *exoU* and 6 *exoS*) isolates from India

| Strain ID | T3SS | *mutL* | *mutS* | *uvrD* |
| --- | --- | --- | --- | --- |
| AI_1323 | *exoS* |  |  | Val556Ile, Ala641Ser, Ser662Asn, Asn666Ser |
| AI_1678 | *exoS* |  | Leu494fs | Val89His |
| AI_3297 | *exoS* | Ala426Thr | Ala187Ser |  |
| AI_3300 | *exoS* | Ala426Thr | Ala187Ser |  |
| PA_2703 | *exoS* |  |  | Gly34fs |
| U804 | *exoS* |  | Ala187Ser |  |
| AI_1294 | *exoU* | Ala391Val,  Ala4Thr |  | Val556Ile, Ala641Ser, Ser662Asn,  Asn666Ser |
| AI_1609 | *exoU* | Ser436Gly |  | Lys344Arg, Ala641Ser, Ser662Asn,  Asn666Ser |
| MGL_95_B5 | *exoU* | Ala391Val,  Ala4Thr | Ala781Thr | Lys344Arg, Ala417Thr, Ser662Asn,  Asn666Ser |
| MGL_146_B9 | *exoU* | Ala391Val,  Ala4Thr | Ala781Thr | Lys344Arg, Ala417Thr, Ser662Asn,  Asn666Ser |
| *P.aeruginosa*-32 | *exoU* | Ala391Val,  Ala4Thr | Ala781Thr | Lys344Arg, Ala417Thr, Ser662Asn,  Asn666Ser |
| *P.aeruginosa*-33 | *exoU* | Ala391Val,  Ala4Thr | Ala781Thr | Lys344Arg, Ala417Thr, Ser662Asn,  Asn666Ser |
| *P.aeruginosa*-36 | *exoU* | Ala391Val,  Ala4Thr | Ala781Thr | Lys344Arg, Ala417Thr, Ser662Asn,  Asn666Ser |
| PA790 | *exoU* | Ala391Val,  Ala4Thr | Ala781Thr | Lys344Arg, Ala417Thr, Ser662Asn,  Asn666Ser |
| VRFPA08 | *exoU* | Ala4Thr | Ala781Thr | Lys344Arg, Val556Ile, Ala641Ser, Ser662Asn, Asn666Ser |
