# Supplementary material for: Comparative study of antibiotic resistance and virulence profiles of exoU and exoS Pseudomonas aeruginosa isolates from urinary tract infections and microbial keratitis in Bangladesh and India
Source: World J Microbiol Biotechnol. 2026 Oct 12;42(10):602. doi: 10.1007/s11274-026-05320-2 (PMC13658543; doi:10.1007/s11274-026-05320-2)
Supplement: Supplementary file 4 [file 11274_2026_5320_MOESM15_ESM.docx]

**Supplementary Table 15**. Distribution of DNA mismatch repair (MMR)-gene SNP burden across sequence types in Bangladeshi and Indian UTI isolates

| Cohort | Isolate | T3SS | ST | *mutL* | *mutS* | *uvrD* | Total Number of SNPs in *mut* genes^1^ | Total number of SNPs in AMR-associated genes^2^ |
| --- | --- | --- | --- | --- | --- | --- | --- | --- |
| Bangladesh | PA331 | *exoS* | ST1221 | 1 | 1 | 0 | 2 | 58 |
| Bangladesh | PA333 | *exoS* | ST244 | 0 | 0 | 0 | 0 | 11 |
| Bangladesh | PA334 | *exoS* | ST362 | 0 | 0 | 4 | 4 | 15 |
| Bangladesh | PA335 | *exoS* | ST244 | 0 | 0 | 0 | 0 | 11 |
| Bangladesh | PA341 | *exoS* | ST1221 | 1 | 1 | 0 | 2 | 58 |
| Bangladesh | PA345 | *exoS* | ST1221 | 1 | 1 | 0 | 2 | 58 |
| Bangladesh | PA348 | *exoS* | ST5190 | 0 | 3 | 1 | 4 | 29 |
| Bangladesh | PA356 | *exoS* | ST360 | 1 | 0 | 0 | 1 | 14 |
| Bangladesh | PA360 | *exoS* | ST1129 | 0 | 0 | 2 | 2 | 20 |
| Bangladesh | PA368 | *exoS* | ST3337 | 0 | 1 | 1 | 2 | 18 |
| Bangladesh | PA371 | *exoS* | ST645 | 0 | 0 | 2 | 2 | 16 |
| Bangladesh | PA373 | *exoS* | ST4217 | 0 | 0 | 2 | 2 | 17 |
| Bangladesh | PA374 | *exoS* | ST260 | 0 | 1 | 2 | 3 | 23 |
| Bangladesh | PA375 | *exoS* | ST260 | 0 | 1 | 2 | 3 | 23 |
| **Bangladesh** | **PA338** | ***exoU*** | **ST4787** | **2** | **1** | **4** | **7** | **32** |
| Bangladesh | PA343 | *exoU* | ST773 | 1 | 0 | 0 | 1 | 25 |
| **Bangladesh** | **PA353** | ***exoU*** | **ST4787** | **2** | **1** | **4** | **7** | **32** |
| Bangladesh | PA357 | *exoU* | ST235 | 1 | 1 | 0 | 2 | 34 |
| **Bangladesh** | **PA362** | ***exoU*** | **ST357** | **1** | **0** | **4** | **5** | **40** |
| **Bangladesh** | **PA364** | ***exoU*** | **ST357** | **1** | **0** | **4** | **5** | **40** |
| **Bangladesh** | **PA367** | ***exoU*** | **ST357** | **1** | **0** | **4** | **5** | **40** |
| **Bangladesh** | **PA372** | ***exoU*** | **ST357** | **1** | **0** | **4** | **5** | **36** |
| Bangladesh | PA376 | *exoU* | ST773 | 1 | 0 | 0 | 1 | 27 |
| India | AI_1323 | *exoS* | ST796 | 0 | 0 | 4 | 4 | 19 |
| India | AI_1678 | *exoS* | ST2629 | 0 | 1 | 1 | 2 | 16 |
| India | AI_3297 | *exoS* | ST988 | 1 | 1 | 0 | 2 | 19 |
| India | AI_3300 | *exoS* | ST988 | 1 | 1 | 0 | 2 | 19 |
| India | PA_2703 | *exoS* | ST244 | 0 | 0 | 1 | 1 | 26 |
| India | U804 | *exoS* | ST654 | 0 | 1 | 0 | 1 | 14 |
| **India** | **AI_1294** | ***exoU*** | **ST308** | **2** | **0** | **4** | **6** | **37** |
| **India** | **AI_1609** | ***exoU*** | **ST357** | **1** | **0** | **4** | **5** | **42** |
| **India** | **MGL_146_B9** | ***exoU*** | **ST1047** | **2** | **1** | **4** | **7** | **38** |
| **India** | **MGL_95_B5** | ***exoU*** | **ST1047** | **2** | **1** | **4** | **7** | **41** |
| **India** | *P. aeruginosa*-32 | ***exoU*** | **ST1047** | **2** | **1** | **4** | **7** | **38** |
| **India** | *P. aeruginosa*-33 | ***exoU*** | **ST1047** | **2** | **1** | **4** | **7** | **38** |
| **India** | *P. aeruginosa*-36 | ***exoU*** | **ST1047** | **2** | **1** | **4** | **7** | **38** |
| **India** | **PA790** | ***exoU*** | **ST773** | **2** | **1** | **4** | **7** | **34** |
| **India** | **VRFPA08** | ***exoU*** | **ST823** | **1** | **1** | **5** | **7** | **39** |

Rows with total SNP counts in *mut* genes ≥5 are shown in **bold** to identify the isolates described in the manuscript as having elevated MMR SNP counts. ^1^ *mut* genes included *mutL, mutS,* and *uvrD*. ^2^AMR-related genes comprised *gyrA, gyrB, parC, parE, nfxB, mexR, nalC, nalD, mexT, mexA, mexB, oprM, mexC, mexD, oprJ, mexE, mexF, oprN, parR,* *parS,* and *fusA2*
