# Supplementary material for: Comparative study of antibiotic resistance and virulence profiles of exoU and exoS Pseudomonas aeruginosa isolates from urinary tract infections and microbial keratitis in Bangladesh and India
Source: World J Microbiol Biotechnol. 2026 Oct 12;42(10):602. doi: 10.1007/s11274-026-05320-2 (PMC13658543; doi:10.1007/s11274-026-05320-2)
Supplement: Supplementary file 5 [file 11274_2026_5320_MOESM1_ESM.docx]

Supplementary Table 1: *P. aeruginosa* UTI strains from Bangladesh and UTI and MK strains from India

| SL. No | Isolates type | Strain ID | T3SS Group | Country | BioProject No |
| --- | --- | --- | --- | --- | --- |
| 1. | UTI isolates | PA331 | *exoS* | Bangladesh | PRJEB110861 |
| 2. |  | PA333 | *exoS* | Bangladesh | PRJEB110861 |
| 3. |  | PA334 | *exoS* | Bangladesh | PRJEB110861 |
| 4. |  | PA335 | *exoS* | Bangladesh | PRJEB110861 |
| 5. |  | PA341 | *exoS* | Bangladesh | PRJEB110861 |
| 6. |  | PA345 | *exoS* | Bangladesh | PRJEB110861 |
| 7. |  | PA348 | *exoS* | Bangladesh | PRJEB110861 |
| 8. |  | PA356 | *exoS* | Bangladesh | PRJEB110861 |
| 9. |  | PA360 | *exoS* | Bangladesh | PRJEB110861 |
| 10. |  | PA368 | *exoS* | Bangladesh | PRJEB110861 |
| 11. |  | PA371 | *exoS* | Bangladesh | PRJEB110861 |
| 12. |  | PA373 | *exoS* | Bangladesh | PRJEB110861 |
| 13. |  | PA374 | *exoS* | Bangladesh | PRJEB110861 |
| 14. |  | PA375 | *exoS* | Bangladesh | PRJEB110861 |
| 15. |  | PA338 | *exoU* | Bangladesh | PRJEB110861 |
| 16. |  | PA343 | *exoU* | Bangladesh | PRJEB110861 |
| 17. |  | PA353 | *exoU* | Bangladesh | PRJEB110861 |
| 18. |  | PA357 | *exoU* | Bangladesh | PRJEB110861 |
| 19. |  | PA362 | *exoU* | Bangladesh | PRJEB110861 |
| 20. |  | PA364 | *exoU* | Bangladesh | PRJEB110861 |
| 21. |  | PA367 | *exoU* | Bangladesh | PRJEB110861 |
| 22. |  | PA372 | *exoU* | Bangladesh | PRJEB110861 |
| 23. |  | PA376 | *exoU* | Bangladesh | PRJEB110861 |
| 24. |  | AI_1678 | *exoS* | India | PRJNA1074546 |
| 25. |  | AI_3297 | *exoS* | India | PRJNA1074546 |
| 26. |  | AI_3300 | *exoS* | India | PRJNA1074546 |
| 27. |  | U804 | *exoS* | India | PRJNA1102609 |
| 28. |  | PA_2703 | *exoS* | India | PRJNA927155 |
| 29. |  | AI_1323 | *exoS* | India | PRJNA1074546 |
| 30. |  | MGL_146_B9 | *exoU* | India | PRJNA924087 |
| 31. |  | MGL_95_B5 | *exoU* | India | PRJNA924087 |
| 32. |  | *P. aeruginosa*-32 | *exoU* | India | PRJNA514245 |
| 33. |  | *P. aeruginosa*-36 | *exoU* | India | PRJNA514245 |
| 34. |  | *P. aeruginosa*-33 | *exoU* | India | PRJNA514245 |
| 35. |  | AI_1294 | *exoU* | India | PRJNA1074546 |
| 36. |  | AI_1609 | *exoU* | India | PRJNA1074546 |
| 37. |  | PA790 | *exoU* | India | PRJNA729627 |
| 38. |  | VRFPA08 | *exoU* | India | PRJNA230869 |
| 39. | Keratitis  isolates | PA188 | *exoS* | India | PRJNA590804 |
| 40. |  | PA189 | *exoS* | India | PRJNA590804 |
| 41. |  | PA193 | *exoS* | India | PRJNA590804 |
| 42. |  | PA206 | *exoS* | India | PRJNA590804 |
| 43. |  | PA216 | *exoS* | India | PRJNA590804 |
| 44. |  | PA218 | *exoS* | India | PRJNA590804 |
| 45. |  | PA31 | *exoU* | India | PRJNA431326 |
| 46. |  | PA32 | *exoU* | India | PRJNA431326 |
| 47. |  | PA33 | *exoU* | India | PRJNA431326 |
| 48. |  | PA34 | *exoU* | India | PRJNA431326 |
| 49. |  | PA35 | *exoU* | India | PRJNA431326 |
| 50. |  | PA37 | *exoU* | India | PRJNA431326 |
| 51. |  | PA82 | *exoU* | India | PRJNA431326 |
| 52. |  | PA198 | *exoU* | India | PRJNA590804 |
| 53. |  | PA202 | *exoU* | India | PRJNA590804 |
| 54. |  | PA217 | *exoU* | India | PRJNA590804 |
| 55. |  | PA219 | *exoU* | India | PRJNA590804 |
| 56. |  | PA220 | *exoU* | India | PRJNA590804 |
| 57. |  | PA221 | *exoU* | India | PRJNA590804 |
