# Supplementary material for: Comparative study of antibiotic resistance and virulence profiles of exoU and exoS Pseudomonas aeruginosa isolates from urinary tract infections and microbial keratitis in Bangladesh and India
Source: World J Microbiol Biotechnol. 2026 Oct 12;42(10):602. doi: 10.1007/s11274-026-05320-2 (PMC13658543; doi:10.1007/s11274-026-05320-2)
Supplement: Supplementary file 6 [file 11274_2026_5320_MOESM2_ESM.docx]

Supplementary Table 2: Antimicrobial susceptibility results of 9 *exoU* and 14 *exoS* *P. aeruginosa* UTI isolates from Bangladesh

| Strain ID | Ciprofloxacin (µg/ml),  Breakpoint ≤1,2, ≥ 4 | Levofloxacin (µg/ml),  Breakpoint ≤2, 4, ≥ 8 | Gentamicin (µg/ml),  Breakpoint ≤4, 8, ≥ 16 | Tobramycin (µg/ml),  Breakpoint ≤4, 8, ≥ 16 |
| --- | --- | --- | --- | --- |
| PA331 | 1 | 2 | 4 | 2 |
| PA333 | 1 | 1 | 4 | 2 |
| PA334 | 1 | 1 | 4 | 2 |
| PA335 | 0.25 | 2 | **16** | 2 |
| PA338 | 0.5 | 1 | 4 | **≥5120** |
| PA341 | 0.5 | 0.5 | 4 | 1 |
| PA343 | **2** | **4** | 4 | **16** |
| PA345 | **16** | **32** | **512** | **512** |
| PA348 | **32** | **32** | **512** | **512** |
| PA353 | **2** | **4** | 4 | 1 |
| PA356 | 1 | 2 | **16** | 2 |
| PA357 | **128** | **128** | **512** | **≥5120** |
| PA360 | **256** | **128** | **32** | **32** |
| PA362 | **1024** | **1024** | **≥5120** | **≥5120** |
| PA364 | **256** | **1024** | **≥5120** | **≥5120** |
| PA367 | **128** | **256** | **512** | **≥5120** |
| PA368 | 0.25 | 0.5 | 1 | 0.5 |
| PA371 | 0.25 | 1 | **16** | 4 |
| PA372 | 0.25 | 2 | **32** | 4 |
| PA373 | 0.25 | 2 | **16** | 2 |
| PA374 | 0.25 | 2 | **16** | 2 |
| PA375 | 1 | 2 | 4 | 2 |
| PA376 | **128** | **256** | **≥5120** | **≥5120** |

**Bold** letter indicates Resistance
