# Supplementary material for: Comparative study of antibiotic resistance and virulence profiles of exoU and exoS Pseudomonas aeruginosa isolates from urinary tract infections and microbial keratitis in Bangladesh and India
Source: World J Microbiol Biotechnol. 2026 Oct 12;42(10):602. doi: 10.1007/s11274-026-05320-2 (PMC13658543; doi:10.1007/s11274-026-05320-2)
Supplement: Supplementary file 7 [file 11274_2026_5320_MOESM3_ESM.docx]

Supplementary Table 3: Functional mutations in *gyrA, gyrB, parC, parE* and efflux pumps regulatory genes of 9 *exoU* and 14 *exoS* *P. aeruginosa* UTI isolates from Bangladesh

| Strain ID | TTSS Group | Cip | Lev | *gyrA* | *gyrB* | *parC* | *parE* | *mexR* | *nalC* | *nalD* | *mexT* | *nfxB* |
| --- | --- | --- | --- | --- | --- | --- | --- | --- | --- | --- | --- | --- |
| PA362 | *exoU* | **1024** | **1024** | Thr83Ile |  | Pro752Thr,  Ser87Leu | Asp533Glu | Val126Glu | Gly71Glu,  Ser209Arg | Val133fs | Gln80fs,  Phe172Ile |  |
| PA364 | *exoU* | **256** | **1024** | Thr83Ile |  | Pro752Thr,  Ser87Leu | Asp533Glu | Val126Glu | Gly71Glu,  Ser209Arg | Val133fs | Gln80fs,  Phe172Ile |  |
| PA360 | *exoS* | **256** | **128** |  | His148Asn | Val646Leu | Asp533Glu |  | Gly71Glu,  Ser209Arg |  | Gln80fs,  Phe172Ile |  |
| PA367 | *exoU* | **128** | **256** | Thr83Ile |  | Pro752Thr,  Ser87Leu | Asp533Glu | Val126Glu | Gly71Glu,  Ser209Arg | Val133fs | Gln80fs  Phe172Ile |  |
| PA376 | *exoU* | **128** | **256** | Thr83Ile |  | Ser87Leu | Asp533Glu | Val126Glu | Gly71Glu,  Ser209Arg |  | Arg40His,  Gln80fs,  Phe172Ile |  |
| PA357 | *exoU* | **128** | **128** | Thr83Ile |  | Ser87Leu | Asp533Glu | Val126Glu | Gly71Glu,  Glu153Gln,  Ser209Arg |  | Pro60Ser,  Gln80fs,  Phe172Ile |  |
| PA348 | *exoS* | **32** | **32** | Ser903Ala,  Ala900Gly,  Asp893Glu,  Gly860Ser,  Val671Ile,  Thr83Ile |  | His262Gln,  Ser87Leu | Glu215Gln | Val126Glu | Gly71Glu,  Ser209Arg | Glu120Asp,  Asp147Glu,  Met157Leu,  Asp187Ala | Gln80fs,  Phe172Ile |  |
| PA345 | *exoS* | **16** | **32** |  |  | Ser331Thr | Asp533Glu | Val126Glu,  Ala103Gly | Gly71Glu,  Gln182Lys,  Leu206Val,  Ser209Arg | Asp204Asn | Glu26Gly,  Pro60Ser,  Gln80fs,  Phe172Ile | His109Tyr |
| PA343 | *exoU* | **2** | **4** |  |  |  | Asp533Glu | Val126Glu | Gly71Glu,  Ser209Arg |  | Arg40His,  Gln80fs,  Phe172Ile |  |
| PA353 | *exoU* | **2** | **4** |  | Ala800fs |  | Asp533Glu |  | Gly71Glu,  Ser209Arg |  | Gln80fs,  Phe172Ile |  |
| PA331 | *exoS* | 1 | 2 |  |  | Ser331Thr | Asp533Glu | Val126Glu, Ala103Gly | Gly71Glu,  Gln182Lys,  Leu206Val,  Ser209Arg | Asp204Asn | Glu26Gly Pro60Ser,  Gln80fs,  Phe172Ile | His109Tyr |
| PA333 | *exoS* | 1 | 1 |  |  |  |  |  |  |  | Pro60Ser,  Gln80fs,  Phe172Ile |  |
| PA334 | *exoS* | 1 | 1 |  |  |  |  |  | Gly71Glu | Thr188Ala | Gln80fs,  Phe172Ile |  |
| PA356 | *exoS* | 1 | 2 |  |  | His262Gln |  |  | Gly71Glu,  Ser209Arg |  | Gln80fs,  Phe172Ile |  |
| PA375 | *exoS* | 1 | 2 | Ser912_Glu913del |  | Val646Leu |  |  | Gly71Glu,  Ser209Arg |  | Gln80fs,  Phe172Ile |  |
| PA338 | *exoU* | 0.5 | 1 |  | Ala800fs |  | Asp533Glu |  | Gly71Glu,  Ser209Arg |  | Gln80fs,  Phe172Ile |  |
| PA341 | *exoS* | 0.5 | 0.5 |  |  | Ser331Thr | Asp533Glu | Val126Glu,  Ala103Gly | Gly71Glu,  Gln182Lys,  Leu206Val,  Ser209Arg | Asp204Asn | Glu26Gly,  Pro60Ser,  Gln80fs,  Phe172Ile | His109Tyr |
| PA335 | *exoS* | 0.25 | 2 |  |  |  |  |  |  |  | Pro60Ser,  Gln80fs,  Phe172Ile |  |
| PA368 | *exoS* | 0.25 | 0.5 |  |  |  | Ile57Val |  | Gly71Glu,  Ser209Arg |  | Gln80fs,  Phe172Ile |  |
| PA371 | *exoS* | 0.25 | 1 |  |  | Val646Leu |  |  | Gly71Glu,  Ser209Arg |  | Pro60Ser,  Gln80fs,  Phe172Ile |  |
| PA372 | *exoU* | 0.25 | 2 |  |  | Pro752Thr | Asp533Glu | Val126Glu | Gly71Glu,  Ser209Arg |  | Gln80fs,  Phe172Ile |  |
| PA373 | *exoS* | 0.25 | 2 |  |  |  |  |  | Gly71Glu,  Ser209Arg |  | Pro60Ser,  Gln80fs,  Phe172Ile |  |
| PA374 | *exoS* | 0.25 | 2 | Ser912_Glu913del |  | Val646Leu |  |  | Gly71Glu,  Ser209Arg |  | Gln80fs,  Phe172Ile |  |

Abbreviations: Cip, ciprofloxacin; Lev, levofloxacin; fs, frameshift mutation; del, deletion.

**Bold** numbers indicate resistance; No SNP in *mexZ*. Red font indicates functional SNPs found in UTI samples of Bangladesh;
