# Supplementary material for: Comparative study of antibiotic resistance and virulence profiles of exoU and exoS Pseudomonas aeruginosa isolates from urinary tract infections and microbial keratitis in Bangladesh and India
Source: World J Microbiol Biotechnol. 2026 Oct 12;42(10):602. doi: 10.1007/s11274-026-05320-2 (PMC13658543; doi:10.1007/s11274-026-05320-2)
Supplement: Supplementary file 8 [file 11274_2026_5320_MOESM4_ESM.docx]

Supplementary Table 4: Functional mutations in efflux pump genes of 9 *exoU* and 14 *exoS* *P. aeruginosa* UTI isolates from Bangladesh

| Strain ID | T3SS | *mexA* | *mexB* | *oprM* | *mexC* | *mexD* | *oprJ* | *mexE* | *mexF* | *oprN* |
| --- | --- | --- | --- | --- | --- | --- | --- | --- | --- | --- |
| PA362 | *exoU* |  | Ser1041Glu,  Val1041Ala |  | Pro383Ser,  Ala383Val,  Ala378Thr,  Ser330Ala,  His310Arg,  Ala277Thr,  Ala262Glu,  Glu251Gln | Ser845Ala,  Thr87Ser | Ala260Val | Ser8Phe,  Ala79Gly,  Ala231Thr | Ala843Thr | Ser13Pro,  Ala410Ser |
| PA364 | *exoU* |  | Ser1041Glu,  Val1041Ala |  | Pro383Ser,  Ala383Val,  Ala378Thr,  Ser330Ala,  His310Arg,  Ala277Thr,  Ala262Glu,  Glu251Gln | Ser845Ala,  Thr87Ser | Ala260Val | Ser8Phe,  Ala79Gly,  Ala231Thr | Ala843Thr | Ser13Pro,  Ala410Ser |
| PA360 | *exoS* |  |  |  | Ser330Ala | Ser845Ala,  Glu257Gln | Gln267Arg,  Met69Val,  Ala14Val |  |  |  |
| PA367 | *exoU* |  | Ser1041Glu,  Val1041Ala |  | Pro383Ser,  Ala383Val,  Ala378Thr,  Ser330Ala,  His310Arg,  Ala277Thr,  Ala262Glu,  Glu251Gln | Ser845Ala,  Thr87Ser | Ala260Val | Ser8Phe,  Ala79Gly,  Ala231Thr | Ala843Thr | Ser13Pro,  Ala410Ser |
| PA376 | *exoU* |  |  |  | Pro383Ser,  Ala383Val,  Ala378Thr,  Ser330Ala,  His310Arg,  Ala277Thr,  Ala262Glu,  Glu251Gln | Ser845Ala,  Thr87Ser |  | Ser8Phe |  | Ser13Pro |
| PA357 | *exoU* |  | Gly957Asp,  Ser1041Glu,  Val1041Ala |  | Pro383Ser,  Ser330Ala,  His310Arg,  Arg76Gln,  Ala31Val | Ile982Val,  Ser845Ala | Gln267Arg,  Met69Val | Glu2Val,  Ser8Phe,  Gln368Arg |  | Ser13Pro |
| PA348 | *exoS* | Lys86Glu |  |  |  | Ser845Ala,  Glu257Gln | Asp68Gly,  Met69Val |  |  |  |
| PA345 | *exoS* |  | Asn248Lys |  | Pro383Ser,  Ala383Val,  Ser330Ala,  Ser313Gly,  His310Arg,  Gln300Arg,  Asp298Ala,  Asp226Glu,  Arg76Gln | Ala1039Thr,  Ser915Ala,  Ser845Ala,  Asn669Asp,  Val660Ile,  Glu650Gln,  Ile610Val,  Val523Ala,  Val364Leu,  Ala155Val | Asp351Ala,  Ser264Asn,  Gly184Ser,  Ser176Gly,  Ala163Thr,  Val146Ile,  Gly120Ser,  Ala30Val | Arg387His,  Ser403Thr | Gln788Arg | Ser13Pro,  Thr34Ser,  Thr275Ser |
| PA343 | *exoU* |  |  |  | Pro383Ser,  Ala383Val,  Ala378Thr,  Ser330Ala,  His310Arg,  Ala277Thr,  Ala262Glu,  Glu251Gln | Ser845Ala,  Thr87Ser |  | Ser8Phe |  | Ser13Pro |
| PA353 | *exoU* |  |  |  | Pro383Ser,  Ser330Ala,  Ser313Gly,  His310Arg,  Ala262Glu | Ser845Ala,  Asn775Lys,  Ala237Thr,  Ala12Ser |  | Ser8Phe |  | Ala4Thr,  Ser13Pro,  Ala410Ser |
| PA331 | *exoS* |  | Asn248Lys |  | Pro383Ser,  Ala383Val,  Ser330Ala,  Ser313Gly,  His310Arg,  Gln300Arg,  Asp298Ala,  Asp226Glu,  Arg76Gln | Ala1039Thr,  Ser915Ala,  Ser845Ala,  Asn669Asp,  Val660Ile,  Glu650Gln,  Ile610Val,  Val523Ala,  Val364Leu,  Ala155Val | Asp351Ala,  Ser264Asn,  Gly184Ser,  Ser176Gly,  Ala163Thr,  Val146Ile,  Gly120Ser,  Ala30Val | Arg387His,  Ser403Thr | Gln788Arg | Ser13Pro,  Thr34Ser,  Thr275Ser |
| PA333 | *exoS* |  |  |  | Pro383Ser,  Ser330Ala,  His310Arg,  Arg76Gln | Ser845Ala | Asp68Gly,  Met69Val |  |  |  |
| PA334 | *exoS* | Ala33Thr |  |  |  | Lys1031Arg,  Ser845Ala,  Glu257Gln |  |  |  |  |
| PA356 | *exoS* |  |  | Ala261Thr | Ser330Ala | Ser845Ala,  Glu257Gln | Gln267Arg,  Met69Val |  |  |  |
| PA375 | *exoS* |  |  |  | Pro383Ser,  Ser330Ala,  His310Arg,  Arg76Gln | Ser845Ala,  Ala536Ser | Asp68Gly,  Met69Val |  | Phe509Val |  |
| PA338 | *exoU* |  |  |  | Pro383Ser,  Ser330Ala,  Ser313Gly,  His310Arg,  Ala262Glu | Ser845Ala,  Asn775Lys,  Ala237Thr,  Ala12Ser |  | Ser8Phe |  | Ala4Thr,  Ser13Pro,  Ala410Ser |
| PA341 | *exoS* |  | Asn248Lys |  | Pro383Ser,  Ala383Val,  Ser330Ala,  Ser313Gly,  His310Arg,  Gln300Arg,  Asp298Ala,  Asp226Glu,  Arg76Gln | Ala1039Thr,  Ser915Ala,  Ser845Ala,  Asn669Asp,  Val660Ile,  Glu650Gln,  Ile610Val,  Val523Ala,  Val364Leu,  Ala155Val | Asp351Ala,  Ser264Asn,  Gly184Ser,  Ser176Gly,  Ala163Thr,  Val146Ile,  Gly120Ser,  Ala30Val | Arg387His,  Ser403Thr | Gln788Arg | Ser13Pro,  Thr34Ser,  Thr275Ser |
| PA335 | *exoS* |  |  |  | Pro383Ser,  Ser330Ala,  His310Arg,  Arg76Gln | Ser845Ala | Asp68Gly,  Met69Val |  |  |  |
| PA368 | *exoS* | Glu318* |  |  | Pro383Ser,  Ser330Ala,  His310Arg | Ser845Ala,  Glu257Gln | Met69Val |  |  |  |
| PA371 | *exoS* |  |  |  |  | Glu257Gln | Gln267Arg,  Met69Val,  Ala14Val |  |  |  |
| PA372 | *exoU* |  | Ser1041Glu  Val1041Ala |  | Pro383Ser,  Ala383Val,  Ala378Thr,  Ser330Ala,  His310Arg,  Ala277Thr,  Ala262Glu,  Glu251Gln | Ser845Ala,  Thr87Ser | Ala260Val | Ser8Phe,  Ala79Gly,  Ala231Thr | Ala843Thr | Ser13Pro,  Ala410Ser |
| PA373 | *exoS* | Asp373Glu |  |  | Ser330Ala | Ser845Ala,  Glu257Gln | Gln267Arg,  Met69Val |  |  | Arg361His |
| PA374 | *exoS* |  |  |  | Pro383Ser,  Ser330Ala,  His310Arg,  Arg76Gln | Ser845Ala,  Ala536Ser | Asp68Gly,  Met69Val |  | Phe509Val |  |

Red font indicates functional mutations found in UTI samples of Bangladesh.

No SNPs were observed in *mexX* and *mexY*, so were not shown in the table.
