# Supplementary material for: Comparative study of antibiotic resistance and virulence profiles of exoU and exoS Pseudomonas aeruginosa isolates from urinary tract infections and microbial keratitis in Bangladesh and India
Source: World J Microbiol Biotechnol. 2026 Oct 12;42(10):602. doi: 10.1007/s11274-026-05320-2 (PMC13658543; doi:10.1007/s11274-026-05320-2)
Supplement: Supplementary file 9 [file 11274_2026_5320_MOESM5_ESM.docx]

Supplementary Table 5: The mutations in fluoroquinolone resistance genes that were not significantly different between 9 *exoU* and 14 *exoS* UTI isolates from Bangladesh

| Gene | Mutation |
| --- | --- |
| *gyrA* | Ala900Gly, Asp893Glu, Gly860Ser, Ser903Ala, Ser912_Glu913del, Val671Ile |
| *gyrB* | Ala800fs, His148Asn |
| *parC* | Pro752Thr, Ser331Thr, Val646Leu |
| *parE* | Glu215Gln, Ile57Val |
| *mexA* | Ala33Thr, Asp373Glu, Glu318Ter, Lys86Glu |
| *mexC* | Ala31Val, Asp226Glu, Asp298Ala, Pro383Ser, Ala384Val, Gln300Arg, Arg76Gln, Ser313Gly, Ser330Ala |
| *mexD* | Ala1039Thr, Ala12Ser, Ala155Val, Ala237Thr, Ala536Ser, Glu650Gln, Ile610Val, Ile982Val, Lys1031Arg, Asn669Asp, Asn775Lys, Ser845Ala, Ser915Ala, Val364Leu, Val523Ala, Val660Ile |
| *mexE* | Glu2Val, Gln368Arg, Arg387His, Ser403Thr |
| *mexF* | Phe509Val, Gln788Arg |
| *mexR* | Ala103Gly |
| *mexT* | Glu26Gly, Phe172Ile, Pro60Ser, Gln80fs, Arg40His |
| *nalC* | Glu153Gln, Gly71Glu, Leu206Val, Gln182Lys, Ser209Arg |
| *nalD* | Asp147Glu, Asp187Ala, Asp204Asn, Glu120Asp, Met157Leu, Thr188Ala |
| *oprJ* | Ala14Val, Ala163Thr, Ala30Val, Asp351Ala, Asp68Gly, Met69Val, Gly120Ser, Gly184Ser, Gln267Arg, Ser176Gly, Ser264Asn, Val146Ile |
| *oprM* | Ala261Thr |
| *oprN* | Ala4Thr, Arg361His, Thr275Ser, Thr34Ser |
| *nfxB* | His109Tyr |

Abbreviations: fs, frameshift mutation; del, deletion; Ter=termination
