# Supplementary material for: Comparative study of antibiotic resistance and virulence profiles of exoU and exoS Pseudomonas aeruginosa isolates from urinary tract infections and microbial keratitis in Bangladesh and India
Source: World J Microbiol Biotechnol. 2026 Oct 12;42(10):602. doi: 10.1007/s11274-026-05320-2 (PMC13658543; doi:10.1007/s11274-026-05320-2)
Supplement: Supplementary file 10 [file 11274_2026_5320_MOESM6_ESM.docx]

Supplementary Table 6. Possession of acquired resistance genes and functional mutations in genes associated with aminoglycoside resistance of 9 *exoU* and 14 *exoS* UTI isolates from Bangladesh

| Strain ID | T3SS | Gentamicin | Tobramycin | *aph(3')-IIb* | *aph(3'')-Ib* | *aph(6)- Id* | *aac(6')-II* | *aac(3)-Ic* | *aac(3)-Id* | *ant(4')-IIb* | *aadA6* | *aadA11* | *rmtB* | *rmtF* | *fusA2* | *parR* | *parS* |
| --- | --- | --- | --- | --- | --- | --- | --- | --- | --- | --- | --- | --- | --- | --- | --- | --- | --- |
| PA362 | *exoU* | **≥5120** | **≥5120** |  |  |  |  |  |  |  |  |  |  |  | Gln161Leu,  Ser176Ala,  Ala197Asp,  Gly695Ala | Ser170Asn,  Leu153Arg | His398Arg,  Ala13Thr |
| PA364 | *exoU* | **≥5120** | **≥5120** |  |  |  |  |  |  |  |  |  |  |  | Gln161Leu,  Ser176Ala,  Ala197Asp,  Gly695Ala | Ser170Asn,  Leu153Arg | His398Arg,  Ala13Thr |
| PA376 | *exoU* | **≥5120** | **≥5120** |  |  |  |  |  |  |  |  |  |  |  | Ser176Ala,  Ala197Asp,  Gly695Ala | Ser170Asn,  Leu153Arg | His398Arg |
| PA357 | *exoU* | **512** | **≥5120** |  |  |  |  |  |  |  |  |  |  |  | Ser176Ala,  Ala197Asp,  Gly695Ala | Ser170Asn,  Leu153Arg | His398Arg,  Ala149Thr |
| PA367 | *exoU* | **512** | **≥5120** |  |  |  |  |  |  |  |  |  |  |  | Gln161Leu,  Ser176Ala,  Ala197Asp,  Gly695Ala | Ser170Asn,  Leu153Arg | His398Arg,  Ala13Thr |
| PA338 | *exoU* | 4 | **≥5120** |  |  |  |  |  |  |  |  |  |  |  | Ser176Ala,  Ala197Asp,  Gly695Ala | Ser170Asn,  Leu153Arg | His398Arg |
| PA345 | *exoS* | **512** | **512** |  |  |  |  |  |  |  |  |  |  |  | Ser176Ala,  Gly695Ala | Leu153Arg,  Thr135Ala | Tyr407His,  His398Arg,  Glu343Asp,  Val304Ile,  Ala115Glu |
| PA348 | *exoS* | **512** | **512** |  |  |  |  |  |  |  |  |  |  |  | Ser176Ala,  Gly695Ala |  | His398Arg |
| PA360 | *exoS* | **32** | **32** |  |  |  |  |  |  |  |  |  |  |  | Ser176Ala,  Lys600Arg,  Gly695Ala |  | His398Arg,  Ala345Thr |
| PA343 | *exoU* | 4 | **16** |  |  |  |  |  |  |  |  |  |  |  | Ser176Ala,  Ala197Asp,  Gly695Ala | Ser170Asn,  Leu153Arg | His398Arg |
| PA372 | *exoU* | **32** | 4 |  |  |  |  |  |  |  |  |  |  |  | Gln161Leu,  Ser176Ala,  Ala197Asp,  Gly695Ala | Ser170Asn,  Leu153Arg | His398Arg |
| PA371 | *exoS* | **16** | 4 |  |  |  |  |  |  |  |  |  |  |  | Ser176Ala,  Gln601His,  Gly695Ala |  | His398Arg |
| PA335 | *exoS* | **16** | 2 |  |  |  |  |  |  |  |  |  |  |  | Gly65Ala |  | His398Arg |
| PA356 | *exoS* | **16** | 2 |  |  |  |  |  |  |  |  |  |  |  | Gly695Ala |  | His398Arg |
| PA373 | *exoS* | **16** | 2 |  |  |  |  |  |  |  |  |  |  |  | Ser176Ala,  Gly695Ala |  | His398Arg |
| PA374 | *exoS* | **16** | 2 |  |  |  |  |  |  |  |  |  |  |  | Ser176Ala,  Gln601His,  Gly695Ala | Ile80Leu,  Val55Ile | His398Arg |
| PA331 | *exoS* | 4 | 2 |  |  |  |  |  |  |  |  |  |  |  | Ser176Ala,  Gly695Ala | Leu153Arg,  Thr135Ala | Tyr407His,  His398Arg,  Glu343Asp,  Val304Ile,  Ala115Glu |
| PA333 | *exoS* | 4 | 2 |  |  |  |  |  |  |  |  |  |  |  | Gly695Ala |  | His398Arg |
| PA334 | *exoS* | 4 | 2 |  |  |  |  |  |  |  |  |  |  |  | Ser176Ala,  Gly695Ala |  | His398Arg |
| PA375 | *exoS* | 4 | 2 |  |  |  |  |  |  |  |  |  |  |  | Ser176Ala,  Gln601His,  Gly695Ala | Ile80Leu,  Val55Ile | His398Arg |
| PA341 | *exoS* | 4 | 1 |  |  |  |  |  |  |  |  |  |  |  | Ser176Ala,  Gly695Ala | Leu153Arg,  Thr135Ala | Tyr407His,  His398Arg,  Glu343Asp,  Val304Ile,  Ala115Glu |
| PA353 | *exoU* | 4 | 1 |  |  |  |  |  |  |  |  |  |  |  | Ser176Ala,  Ala197Asp,  Gly695Ala | Ser170Asn,  Leu153Arg | His398Arg |
| PA368 | *exoS* | 1 | 0.5 |  |  |  |  |  |  |  |  |  |  |  | Ser176Ala,  Gly695Ala |  | His398Arg,  Ala345Thr |

Red indicates functional mutations; **Bold** numbers indicate resistance; Dark Box indicates presence of acquired resistance gene.
