# Supplementary material for: Comparative study of antibiotic resistance and virulence profiles of exoU and exoS Pseudomonas aeruginosa isolates from urinary tract infections and microbial keratitis in Bangladesh and India
Source: World J Microbiol Biotechnol. 2026 Oct 12;42(10):602. doi: 10.1007/s11274-026-05320-2 (PMC13658543; doi:10.1007/s11274-026-05320-2)
Supplement: Supplementary file 11 [file 11274_2026_5320_MOESM7_ESM.docx]

Supplementary Table 7: Mutations in the DNA mismatch repair (MMR) system genes of 23 UTI isolates (9 *exoU* and 14 *exoS* ) from Bangladesh

| Strain ID | T3SS | *mutL* | *mutS* | *uvrD* |
| --- | --- | --- | --- | --- |
| PA331 | *exoS* | Ser2Asn | Ser281Gly |  |
| PA333 | *exoS* |  |  |  |
| PA334 | *exoS* |  |  | Gly108Arg, Ser341Arg, Ser662Asn, Asn666Ser |
| PA335 | *exoS* |  |  |  |
| PA338 | *exoU* | Ala391Val, Ala4Thr | Ala781Thr | Lys344Arg, Ala641Ser, Ser662Asn, Asn666Ser |
| PA341 | *exoS* | Ser2Asn | Ser281Gly |  |
| PA343 | *exoU* | Ser2Asn |  |  |
| PA345 | *exoS* | Ser2Asn | Ser281Gly |  |
| PA348 | *exoS* |  | Thr593Ser, Ala781Ser, Met803Val | Asp476Asn |
| PA353 | *exoU* | Ala391Val, Ala4Thr | Ala781Thr | Lys344Arg, Ala641Ser, Ser662Asn, Asn666Ser |
| PA356 | *exoS* | Glu428Asp |  |  |
| PA357 | *exoU* | Ala4Thr | Ala781Thr |  |
| PA360 | *exoS* |  |  | Ser662Asn, Asn666Ser |
| PA362 | *exoU* | Ser436Gly |  | Lys344Arg, Ala641Ser, Ser662Asn,  Asn666Ser |
| PA364 | *exoU* | Ser436Gly |  | Lys344Arg, Ala641Ser, Ser662Asn,  Asn666Ser |
| PA367 | *exoU* | Ser436Gly |  | Lys344Arg, Ala641Ser, Ser662Asn, Asn666Ser |
| PA368 | *exoS* |  | Ala781Thr | Thr547Ala |
| PA371 | *exoS* |  |  | Ser662Asn, Asn666Ser |
| PA372 | *exoU* | Ser436Gly |  | Lys344Arg, Ala641Ser, Ser662Asn, Asn666Ser |
| PA373 | *exoS* |  |  | Ser662Asn,Asn666Ser |
| PA374 | *exoS* |  | Ala781Thr | Ser662Asn, Asn666Ser |
| PA375 | *exoS* |  | Ala781Thr | Ser662Asn,Asn666Ser |
| PA376 | *exoU* | Ser2Asn |  |  |
