# Supplementary material for: Comparative study of antibiotic resistance and virulence profiles of exoU and exoS Pseudomonas aeruginosa isolates from urinary tract infections and microbial keratitis in Bangladesh and India
Source: World J Microbiol Biotechnol. 2026 Oct 12;42(10):602. doi: 10.1007/s11274-026-05320-2 (PMC13658543; doi:10.1007/s11274-026-05320-2)
Supplement: Supplementary file 12 [file 11274_2026_5320_MOESM8_ESM.docx]

Supplementary Table 8: Functional mutations in *gyrA, gyrB, parC, parE* and efflux pumps regulatory genes and possession of *qnrVC1* gene of 9 *exoU* and 6 *exoS* *P. aeruginosa* UTI isolates from India

| **Strain ID** | **T3SS** | ***gyrA*** | ***gyrB*** | ***parC*** | ***parE*** | ***mexR*** | ***nalC*** | ***nalD*** | ***mexT*** | ***nfxB*** | ***qnrVC1*** |
| --- | --- | --- | --- | --- | --- | --- | --- | --- | --- | --- | --- |
| AI_1323 | *exoS* |  |  | Val646Leu | Asp533Glu | Val126Glu | Gly71Glu, Ser209Arg |  | Pro60Ser, Gln80fs |  |  |
| AI_1678 | *exoS* |  |  |  | Asp142Asn |  | Gly71Glu,  Ala186Thr |  | Pro60Ser,  Gln80fs,  Phe172Ile |  |  |
| AI_3297 | *exoS* |  |  | Val646Leu |  |  | Gly71Glu,  Ser209Arg |  | Pro60Ser,  Gln80fs,  Phe172Ile |  |  |
| AI_3300 | *exoS* |  |  | Val646Leu |  |  | Gly71Glu,  Ser209Arg |  | Pro60Ser,  Gln80fs,  Phe172Ile |  |  |
| PA_2703 | *exoS* | Ser903Ala,  Ala900Gly,  Asp893Glu,  Gly860Ser,  Val671Ile,  Tyr100Cys,  Thr83Ile |  | Ser87Leu | Val125fs |  |  | Val151fs | Pro60Ser,  Gln80fs,  Phe172Ile |  |  |
| U804 | *exoS* | Thr83Ile | Glu468Asp | Ser87Leu,  Ala88Pro, |  |  | Gly71Glu | Leu153Gln | Pro60Ser,  Phe172Ile |  |  |
| AI_1294 | *exoU* | Thr83Ile |  | Pro752Thr,  Ser87Leu | Asp533Glu | Val126Glu | Gly71Glu,  Asp79Glu,  Ser209Arg | Gly81fs | Gln80fs,  Phe172Ile |  |  |
| AI_1609 | *exoU* | Thr83Ile |  | Pro752Thr,  Ser87Leu | Asp533Glu | Val126Glu | Gly71Glu,  Ser209Arg | Val133fs | Gln80fs,  Phe172Ile |  |  |
| MGL_95_B5 | *exoU* | Thr83Ile |  | Asp645Gly,  Ala639Gly,  Ser87Leu | Asp533Glu | Val126Glu | Ser5fs,  Gly71Glu,  Ser209Arg,  Gly213Asp |  | Pro60Ser,  Gln80fs,  Phe172Ile |  |  |
| MGL_146_B9 | *exoU* | Thr83Ile |  | Ser87Leu | Asp533Glu | Val126Glu | Ser5fs,  Gly71Glu,  Ser209Arg,  Gly213Asp |  | Pro60Ser,  Gln80fs,  Phe172Ile |  |  |
| *P.aeruginosa*-32 | *exoU* | Thr83Ile |  | Ser87Leu | Asp533Glu | Val126Glu | Ser5fs,  Gly71Glu,  Ser209Arg,  Gly213Asp |  | Pro60Ser,  Gln80fs,  Phe172Ile |  |  |
| *P.aeruginosa*-33 | *exoU* | Thr83Ile |  | Ser87Leu | Asp533Glu | Val126Glu | Ser5fs,  Gly71Glu,  Ser209Arg,  Gly213Asp |  | Pro60Ser,  Gln80fs,  Phe172Ile |  |  |
| *P.aeruginosa*-36 | *exoU* | Thr83Ile |  | Ser87Leu | Asp533Glu | Val126Glu | Ser5fs,  Gly71Glu,  Ser209Arg,  Gly213Asp |  | Pro60Ser,  Gln80fs,  Phe172Ile |  |  |
| PA790 | *exoU* | Thr83Ile |  | Ser87Leu | Asp533Glu | Val126Glu | Gly71Glu,  Ser209Arg |  | Arg40His,  Gln80fs,  Phe172Ile |  |  |
| VRFPA08 | *exoU* | Thr83Ile |  | Ser87Leu | Asp533Glu | Val126Glu | Gly71Glu,  Ser209Arg |  | Gln80fs,  Ala143Thr,  Phe172Ile | Ser5Phe |  |

**Red** indicates functional mutations found in UTI isolates, No SNP in *mexZ*. Abbreviations: fs, frameshift mutation

Dark Box indicates presence of acquired resistance gene.
