# Supplementary material for: Comparative study of antibiotic resistance and virulence profiles of exoU and exoS Pseudomonas aeruginosa isolates from urinary tract infections and microbial keratitis in Bangladesh and India
Source: World J Microbiol Biotechnol. 2026 Oct 12;42(10):602. doi: 10.1007/s11274-026-05320-2 (PMC13658543; doi:10.1007/s11274-026-05320-2)
Supplement: Supplementary file 13 [file 11274_2026_5320_MOESM9_ESM.docx]

**Supplementary Table 9:** Functional mutations in efflux pump genes of 9 *exoU* and 6 *exoS* UTI isolates from India

| **Country** | **Strain ID** | **T3SS** | ***mexA*** | ***mexB*** | ***oprM*** | ***mexC*** | ***mexD*** | ***oprJ*** | ***mexE*** | ***mexF*** | ***oprN*** |
| --- | --- | --- | --- | --- | --- | --- | --- | --- | --- | --- | --- |
| India | **AI_1323** | *exoS* | Asp373Asn |  |  | Ser330Ala | Ser845Ala |  | Ser8Phe |  | Asp31Val |
| India | **AI_1678** | *exoS* |  |  |  |  |  |  |  |  |  |
| India | **AI_3297** | *exoS* |  |  |  | Ser330Ala | Ser845Ala,  Glu257Gln | Arg210His,  Met69Val |  |  | Ala38Thr |
| India | **AI_3300** | *exoS* |  |  |  | Ser330Ala | Ser845Ala,  Glu257Gln | Arg210His,  Met69Val |  |  | Ala38Thr |
| India | **PA_2703** | *exoS* |  |  | Ala309fs | Pro383Ser,  Ser330Ala,  His310Arg,  Arg76Gln | Ser845Ala | Asp68Gly,  Met69Val |  | Ala550Val,  Glu1008fs |  |
| India | **U804** | *exoS* |  |  |  | Ser330Ala | Ser845Ala,  Glu257Gln | Met69Val |  |  |  |
| India | **AI_1294** | *exoU* |  | Gly957Asp,  Ser1041Glu,  Val1041Ala |  | Pro383Ser, Ala384Val,  Ala378Thr,  Ser330Ala,  His310Arg,  Ala277Thr,  Ala262Glu,  Glu251Gln | Ser845Ala,  Thr87Ser |  |  |  | Ser13Pro |
| India | **AI_1609** | *exoU* |  | Ser1041Glu,  Val1041Ala |  | Pro383Ser, Ala384Val,  Ala378Thr,  Ser330Ala,  His310Arg,  Ala277Thr,  Ala262Glu  Glu251Gln | Ser845Ala  Thr87Ser | Ala260Val | Ser8Phe,  Ala79Gly,  Ala231Thr | Ala843Thr | Ser13Pro,  Ala410Ser |
| India | **MGL_95_B5** | *exoU* |  | Ser1041Glu,  Val1041Ala |  | Pro383Ser,  Ser330Ala,  Ala262Glu,  Arg76Gln | Lys1031Arg,  Ser845Ala,  Val434Ala,  Glu257Gln | Thr25Ile | Ser8Phe |  | Ser13Pro,  Val402Gly,  Ser403Pro |
| India | **MGL_146_B9** | *exoU* |  | Ser1041Glu,  Val1041Ala |  | Pro383Ser,  Ser330Ala,  Ala262Glu,  Arg76Gln | Lys1031Arg,  Ser845Ala,  Val434Ala,  Glu257Gln | Thr25Ile | Ser8Phe |  | Ser13Pro |
| India | ***P.aeruginosa-*32** | *exoU* |  | Ser1041Glu,  Val1041Ala |  | Pro383Ser,  Ser330Ala,  Ala262Glu,  Arg76Gln | Lys1031Arg,  Ser845Ala,  Val434Ala,  Glu257Gln | Thr25Ile | Ser8Phe |  | Ser13Pro |
| India | ***P.aeruginosa*-33** | *exoU* |  | Ser1041Glu,  Val1041Ala |  | Pro383Ser,  Ser330Ala,  Ala262Glu,  Arg76Gln | Lys1031Arg,  Ser845Ala,  Val434Ala,  Glu257Gln | Thr25Ile | Ser8Phe |  | Ser13Pro |
| India | ***P.aeruginosa*-36** | *exoU* |  | Ser1041Glu,  Val1041Ala |  | Pro383Ser,  Ser330Ala,  Ala262Glu,  Arg76Gln | Lys1031Arg,  Ser845Ala,  Val434Ala,  Glu257Gln | Thr25Ile | Ser8Phe |  | Ser13Pro |
| India | **PA790** | *exoU* |  |  |  | Pro383Ser, Ala384Val,  Ala378Thr,  Ser330Ala,  His310Arg,  Ala277Thr,  Ala262Glu,  Glu251Gln | Ser845Ala,  Thr87Ser |  | Ser8Phe |  | Ser13Pro |
| India | **VRFPA08** | *exoU* |  |  |  | Pro383Ser,  Glu347Lys,  Ser330Ala,  His310Arg,  Thr175Ala,  Arg76Gln | Ser845Ala,  Thr286Met | Arg168Trp | Ser8Phe,  Asp353Glu,  Asp370Glu | Asp230Ala,  Asp666Glu | Ser13Pro |

All mutations in red indicates functional mutations; no mutations were observed in *mexX* and *mexY;* Abbreviations: fs, frameshift mutation
